# MXene Hollow Spheres Supported by a C–Co Exoskeleton Grow MWCNTs for Efficient Microwave Absorption

**DOI:** 10.1007/s40820-024-01326-3

**Published:** 2024-02-02

**Authors:** Ze Wu, Xiuli Tan, Jianqiao Wang, Youqiang Xing, Peng Huang, Bingjue Li, Lei Liu

**Affiliations:** School of Mechanical Engineering, Southeast University, Nanjing, 211189 People’s Republic of China

**Keywords:** MXene, C–Co skeleton, MWCNTs, Microwave absorption

## Abstract

High-performance microwave absorption (MA) materials must be studied immediately since electromagnetic pollution has become a problem that cannot be disregarded. A straightforward composite material, comprising hollow MXene spheres loaded with C–Co frameworks, was prepared to develop multiwalled carbon nanotubes (MWCNTs). A high impedance and suitable morphology were guaranteed by the C–Co exoskeleton, the attenuation ability was provided by the MWCNTs endoskeleton, and the material performance was greatly enhanced by the layered core–shell structure. When the thickness was only 2.04 mm, the effective absorption bandwidth was 5.67 GHz, and the minimum reflection loss (RL_min_) was − 70.70 dB. At a thickness of 1.861 mm, the sample calcined at 700 °C had a RL_min_ of − 63.25 dB. All samples performed well with a reduced filler ratio of 15 wt%. This paper provides a method for making lightweight core–shell composite MA materials with magnetoelectric synergy.

**Supplementary Information:**

The online version contains supplementary material available at 10.1007/s40820-024-01326-3.

## Highlights

- A hollow core–shell structure was constructed with C–Co as the exoskeleton to support the MXene and multiwalled carbon nanotubes (MWCNTs) endoskeleton, with MWCNTs growing toward the center of the sphere.
- A reflection loss of − 70.70 dB and an effective absorption bandwidth of 5.67 GHz were obtained when the thickness was only 2.04 mm. The powder filler ratio was only 15 wt%.
- The unique hollow core–shell structure enhanced multiple reflection and scattering losses.

**Supplementary Information:**

The online version contains supplementary material available at 10.1007/s40820-024-01326-3.

## Graphical abstract

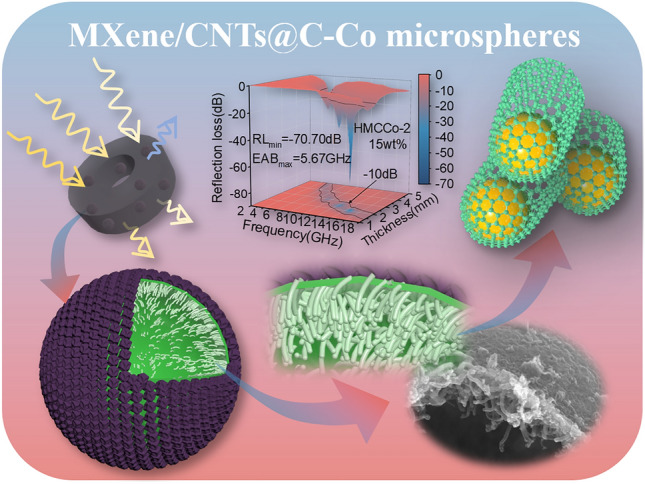

## Introduction

Electromagnetic waves (EMW) have been widely used as functional carriers for information transmission in fields such as communications, medical treatment, and the military thanks to the rapid development of wireless communication technology and the electronic industry, especially with gradual popularization of fifth-generation mobile communication technology (5G) and the proposed 6G [1–9]. In addition to serving humanity, these information carriers also seriously affect electromagnetic safety [10]. As a result, there is a critical need to create effective microwave absorbing materials (MAM), especially in the gigahertz radar frequency band, to extend the lives of electronic components, safeguard the environment and public health, and provide vital information security [11–13].

Excellent MAM design, as is generally known, results from taking into account the composition and microstructure design [14, 15]. Since it is challenging to combine impedance matching and attenuation capability in traditional single components, the MA performance is typically improved via the synergistic effects of many components [16]. For example, some researchers used the pore structures of natural materials to obtain high-performance electromagnetic wave-resistant materials through carbonization and chemical modification [17–19]. Others have constructed structures with large pores to enhance the electromagnetic properties of the material [20–22]. In addition, foldable, flexible, and multifunctional composite materials can be obtained by introducing polymers [23, 24]. Conductive carbon-based materials such as graphene, carbon nanotubes (CNTs), and fibrous carbon materials have received widespread attention due to their high dielectric properties and simple preparations [25, 26]. MXene, a developing two-dimensional graphene-like material, is highly desired in the MA industry because of its high specific surface area, strong conductivity, and abundance of surface functional groups (–F, –O, –OH) [27, 28]. However, high conductivity can quickly lead to a mismatch in the MXene impedance, which can cause incident electromagnetic waves to reflect into the atmosphere [29, 30]. Based on this, researchers have added magnetic materials to the two-dimensional accordion structures of MXene to balance the high conductivity and achieve efficient MA via magnetoelectric synergy [31–34]. Nevertheless, the attenuation capacities of composite MAMs are still somewhat impacted by strongly self-stacking MXene [35, 36]. Therefore, several MXene morphological forms, including aerogels and core–shell structures, have been studied. Due to the numerous heterogeneous interfaces, anisotropy, and distinctive cavities, core–shell structures exhibit efficient EMW attenuation. The core–shell construction also addresses the issue of material self-stacking [14, 37–39]. Currently, core–shell structure construction methods are roughly divided into surface polymerization and wrapping, direct chemical precipitation, self-assembly, and template methods. Among them, the hard template method can be applied by adjusting the template size to regulate the sizes of the nanoparticles [40, 41].

Magnetic particles can be obtained by carbonizing metal–organic frameworks (MOFs). MOFs are crystalline porous materials comprised of metal ions or clusters and organic ligands that provide a designable composition, customizable pore structure, high crystallinity, and a large specific surface area [42]. As a result, they are widely used in water pollution treatment, catalysis, electronics, capacitors, drug delivery, and optics [43–47]. MOFs have gained attention as matrixes in MAM construction since the proposal of carbon-based MAMs produced from Prussian blue in 2015 [48]. Zeolitic imidazolate frameworks (ZIFs) are a class of MOFs materials. ZIF67 is one of the structural materials of ZIFs. Since ZIF67 has a unique structure of rhombic dodecahedron and is simple to prepare, it has been exploited extensively. By carbonizing ZIF67-based materials, multiple polarizations and a distinctive magnetoelectric coordination mechanism were produced, which optimized the electromagnetic parameters and improved the MA performance of the composite material [25, 42]. On the other hand, the carbonized structural morphology, graphitization level, and magnetism of ZIF67 itself are influenced by temperature and other factors. In situ-produced CNTs have been used by some researchers, while others have demonstrated that ZIF67 can produce porous C/Co frameworks with their original architectures after high-temperature calcination [49–52]. It is vital to maintain the outstanding conductivities and excellent aspect ratios of CNTs/MWCNTs, which offer extensive pathways for electron migration and transitions within the MAMs to release electromagnetic energy as thermal energy [53–56].

Based on the foregoing, an innovative strategy for preparing composite core–shell MAMs with MXene (Ti_3_C_2_T_*x*_) as the intermediate layer was developed. The MXene nanosheets were loaded onto a rigid, 0-dimensional template for high-temperature pyrolysis to avoid the stacking issue. The surface functional groups of MXene spheres were utilized as Co^2+^ ion sites, and an entire ZIF67-coated core–shell structure was obtained by self-assembly. The C–Co skeleton produced after high-temperature calcination served as the exoskeleton to support the hollow MXene spheres while maximizing impedance matching. Nonzero-dimensional structures, complex components, and solvent-soluble polymers were excluded from use as sacrificial templates [57–60]. Taken together, and to meet the demand for production of carbon nanotubes, PS microspheres, which are simple and easily accessible, were chosen as templates. The original endoskeleton was replaced by in situ growth of MWCNTs through high-temperature calcination, leading to a successful core–shell structure comprising hollow MXene sphere weaved MWCNTs with a C–Co skeleton (HMCCo). Such a distribution of carbon nanotubes growth on the inner side of the spherical shell has not been reported as far as our knowledge. In addition, for the same materials in the MXene and CNTs systems, the prepared absorbers have ultrahigh MA performance at lower thickness and packing ratios.

## Experimental and Calculation Methods

### Materials

Polystyrene microspheres (PSs, diameter: ~ 10 μm) were obtained from Dongwan Tesu Lang Chemical Raw Material Factory. Ti_3_AlC_2_ powder was purchased from Jilin 11 Technology Co., Ltd. (Jilin, China). Cobalt nitrate hexahydrate (Co(NO_3_)_2_·6H_2_O), 2-methylimidazole, anhydrous methanol, and poly dimethyl diallyl ammonium chloride (PDDA, 20 wt% in H_2_O) solution were obtained from Sinopharm Chemical Reagent Co., Ltd. All materials were used without further purification.

### Synthesis of the MXene/MWCNTs@C–Co Composite

#### Synthesis of PS@MXene

A Ti_3_C_2_T_*x*_ MXene solution was prepared according to a previously reported method [61]. PS was ultrasonically dispersed in 1 wt% PDDA solution and stirred for 12 h. Ultrasonication was carried out in an ice-water bath at 240 w power for 1.5 h. Then, the P-PS white precipitate was collected by centrifugation, washed with deionized water to remove excess PDDA, and finally dried for 24 h in a blast dryer at 70 °C. The obtained P-PS powder was dispersed in deionized water at a concentration of 10 mg mL^−1^, and then it was slowly added to an equal volume of stirred MXene solution (1.0 mg mL^−1^). The mixed solution was stirred and reacted at room temperature for 12 h, followed by centrifugation to obtain a precipitate, and finally, vacuum-dried overnight at 60 °C to obtain PS@MXene (PM).

#### Synthesis of PS@MXene@ZIF67

PS@MXene@ZIF67 (PMZ) was synthesized via a simple method. Co(NO_3_)_2_·6H_2_O (400 mg) and 2-methylimidazole (452 mg) were separately dissolved in 50 mL of anhydrous methanol. PM (*x* mg) was added to the Co(NO_3_)_2_·6H_2_O methanol solution, sonicated at 120 w power for 30 s, and stirred for 2 h. Afterward, the 2-methylimidazole methanol solution was quickly added, and the solution was slowly stirred at room temperature for 24 h. Finally, the purple product was collected by centrifugation and dried in a 60 °C vacuum oven for 6 h. The samples prepared with *x* equal 400, 300, 200, and 100 were denoted as PMZ-1, PMZ-2, PMZ-3, and PMZ-4, respectively.

#### Synthesis of Hollow MXene/MWCNTs@C–Co Microspheres

Hollow MXene/MWCNTs@ C–Co microspheres (HMCCo) were obtained with a high-temperature heat treatment under nitrogen in a tube furnace. PMZ was added to the porcelain boat and placed in a tube furnace. The heating rate of the tube furnace was set at 5 °C min^−1^, the insulation time was 2 h, and the gas flow rate was 80 mL min^−1^. The products formed from PMZ-1, PMZ-2, PMZ-3, and PMZ-4 after heat treatment at 600 °C were labeled as HMCCo-1, HMCCo-2, HMCCo-3, and HMCCo-4, respectively. The heat treatment products of PMZ formed at 400, 500, 600, 700, and 800 °C were labeled HMCCo-400, HMCCo-500, HMCCo-600, HMCCo-700, and HMCCo-800, respectively.

### Characterization

Microscopic morphologies and structures were observed with transmission electron microscopy (TEM, G220, FEI) and scanning electron microscopy (SEM, FEI Inspect F50). The crystal structures of the composite materials were obtained with X-ray diffraction (XRD, Ultima IV, Rigaku). The chemical compositions and elemental valence states were characterized with Raman spectroscopy (RAM-PRO-785E, Agiltron) and X-ray photoelectron spectroscopy (XPS, Thermo ESCALAB 250XI). Hysteresis loops were measured with a vibrating sample magnetometer (VSM, LakeShore7404). Thermogravimetric properties were obtained using a thermogravimetric analyzer (TG209 F3). The prepared HMCCo powder was mixed with paraffin and pressed into a coaxial ring with an outer diameter of 7 mm and an inner diameter of 3.05 mm. The filler ratio of HMCCo in each coaxial ring was 15 wt%. The pre-experimental results for the selection of filler ratio are shown in Fig. S22. The electromagnetic parameters of the sample preparation were based on the coaxial-line method and obtained through a vector network analyzer (Ceyear 3656D) within the frequency range 2–18 GHz. All samples were examined at least three times. Based on this, according to transmission line theory, the reflection loss (RL) was calculated as follows:

1 $${\text{RL}}=20 {\text{log}}|({Z}_{{\text{in}}}-{Z}_{0})/({Z}_{{\text{in}}}+{Z}_{0})|$$

2 $${Z}_{{\text{in}}}={Z}_{0}\sqrt{{\mu }_{r}/{\varepsilon }_{r}}{\text{tanh}}(j\frac{2\pi fd}{c}\sqrt{{\mu }_{r}{\varepsilon }_{r}})$$

where ${Z}_{{\text{in}}}$ and ${Z}_{0}$ are the input impedance of the microwave absorber and free space impedance, ${\mu }_{r}$ is the complex permeability, ${\varepsilon }_{r}$ is the complex permittivity, $f$ is the frequency of the electromagnetic wave, $d$ is the thickness of the absorber, and $c$ is the velocity of light in a vacuum [29, 62].

## Results and Discussion

### Fabrication and Characterization of HMCCo

**Fig. 1:**
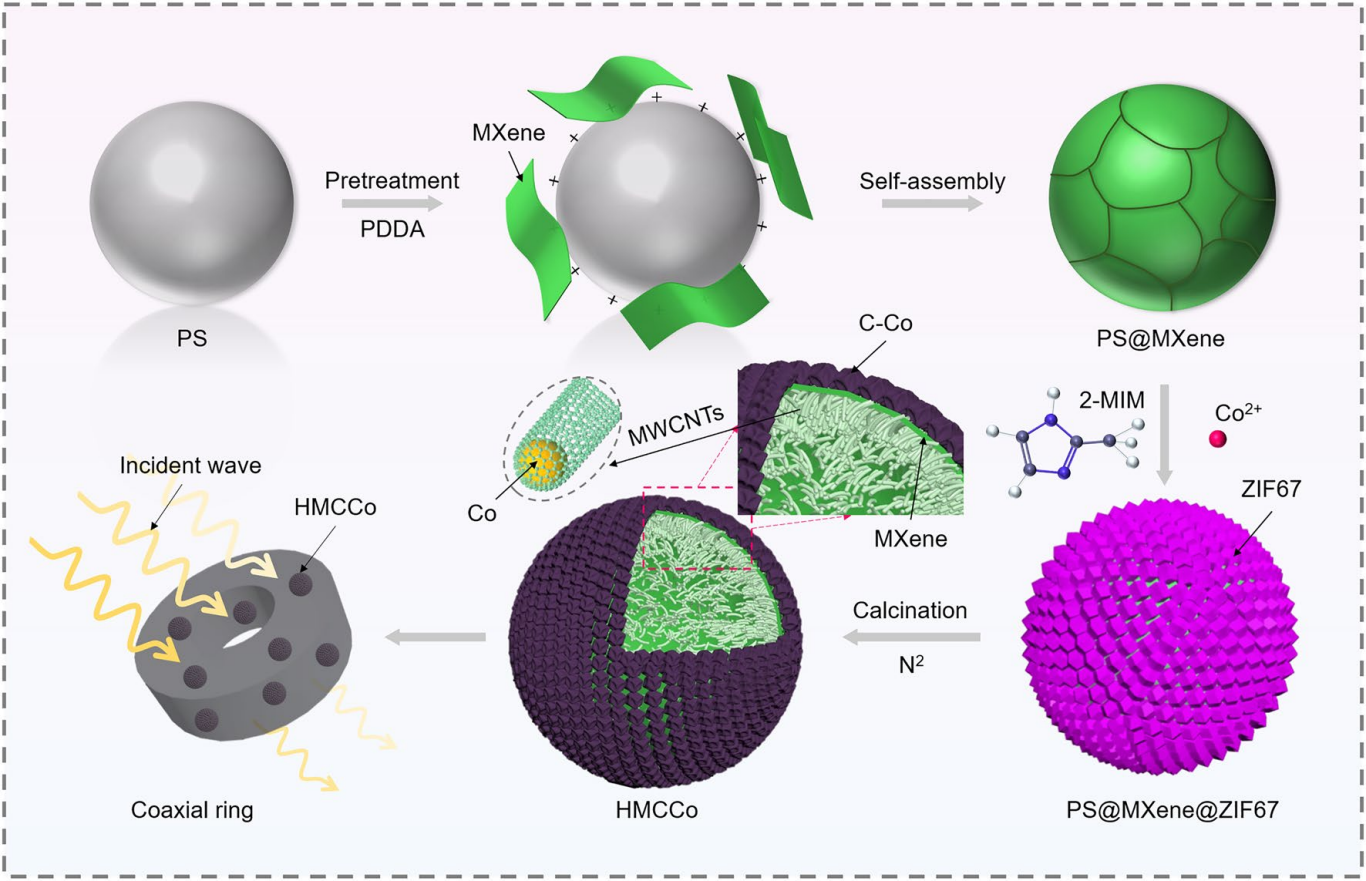
Schematic illustration of the synthetic process for the HMCCo absorber

The fabrication procedures of HMCCo for a hollow sphere structure absorber are shown in Fig. 1. Briefly, few-layer MXene nanosheets were obtained by HCL-LiF etching and ultrasound irradiation. The PS microspheres were positively charged after they were modified with PDDA. After that, the negatively charged MXene nanosheets in the suspension were electrostatically self-assembled onto PDDA-PS spheres through hydrogen bonding. Co^2+^ was captured by the abundant functional groups on MXene and served as the binding sites for subsequent reactions. Then, ZIF67 was self-assembled onto the PS@MXene spheres. Finally, HMCCo was obtained by high-temperature calcination to remove the PS templates. Additionally, the labels and abbreviations used are listed in Table S1.

**Fig. 2:**
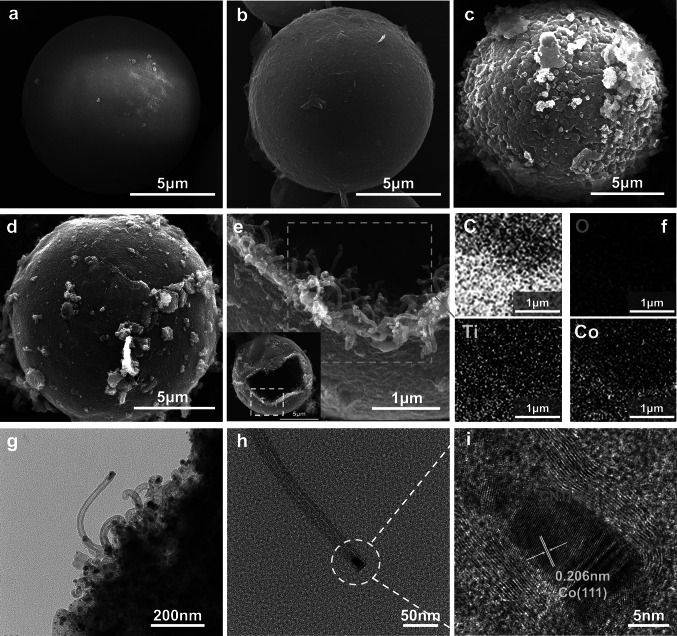
SEM images of **a** PS, **b** PS@MXene, **c** PS@MXene@ZIF67, and **d** HMCCo-2. **e** SEM images of an open HMCCo-2 spherical shell and the internal structure of the spherical shell. **f** Elemental mappings of HMCCo-2. **g** TEM image of the edge of the sample fragments obtained after ultrasonication, including carbon nanotubes grown in situ. **h** TEM image of a single carbon nanotube separated by ultrasound. **i** HRTEM image corresponding to the white circle in (**h)**

The anticipated morphology was revealed by SEM and TEM. As shown in Fig. 2a, the original PS spheres were smooth. In Fig. 2b, MXene nanosheets were successfully and uniformly covered on the surfaces of the PS spheres through electrostatic attraction. Figure 2c shows that the spherical surface was completely wrapped by ZIF67 polyhedral particles with distinct edges and corners. However, if the PS sphere was not coated with MXene nanosheets, the absence of Co^2+^ attachment sites hindered the growth of ZIF67 on the sphere surface, as depicted in Fig. S1. The SEM image (Fig. 2d) of the sample after high-temperature calcination still showed a complete spherical structure, but the ZIF67 on the surface was carbonized and collapsed, resulting in cross-linking. The aforementioned conditions arose for two reasons: on the one hand, during calcination, the organic ligands decomposed; on the other hand, the transition metal cobalt used as a catalyst for formation of the MWCNTs was consumed [63]. In addition, the ZIF67 exoskeleton maintained the original appearance of the spherical shells and prevented them from shrinking after the loss of the PS template at high temperatures. After calcination, a very interesting morphology appeared, with many carbon nanotubes growing toward the interiors of the spherical shells and forming a unique conductive network. This may have occurred because, during calcination, the innermost carbon PS spheres and intermediate layer MXene nanosheets of the spherical shell were rich carbon sources for generation of the carbon nanotubes, while the ZIF67 on the PMZ surface provided sufficient catalyst, resulting in the interesting layer-by-layer assembly. EDS mappings (Fig. 2f) revealed that Co and C were tightly bound and evenly distributed on the MXene.

The edges of the spherical shell fragments grew clear CNTs (Fig. 2g). The separated single CNTs were obtained by ultrasonic (Ice bath, ultrasound at 320 w power for 20 min) dispersion in HMCCo-ethanol, as shown in Figs. 2h and S2. Multiwalled carbon nanotubes with diameters of approximately 15 nm appeared in the high-resolution TEM image (Fig. 2i), and nanoparticles with diameters of approximately 10 nm were encapsulated at the tops of the nanotubes, which was consistent with the SEM image. The spacing of 0.206 nm corresponded to the (111) plane of the Co metal particles, and the outer layer with a spacing of 0.35 nm corresponded to the (002) plane of graphitized carbon [64, 65]. Figure S2b shows the distribution of C and Co, with Co concentrated at the closed top of the CNT. Figure S3 shows the SEM images of PMZ samples made with different mass ratios before and after calcination at 600 ℃. Figure S3a2, b2 shows that when the mass ratio of PS@MXene to cobalt nitrate was 1:2, ZIF67 had a very good coating on the sphere, and the sphere was intact after calcination. When the growth of ZIF67 increased, it was difficult to ensure the integrity of the spheres after calcination. When the mass ratio was 1:4 (Fig. S3a4), the PMZ showed excessive ZIF67 encapsulation and slight detachment.

**Fig. 3:**
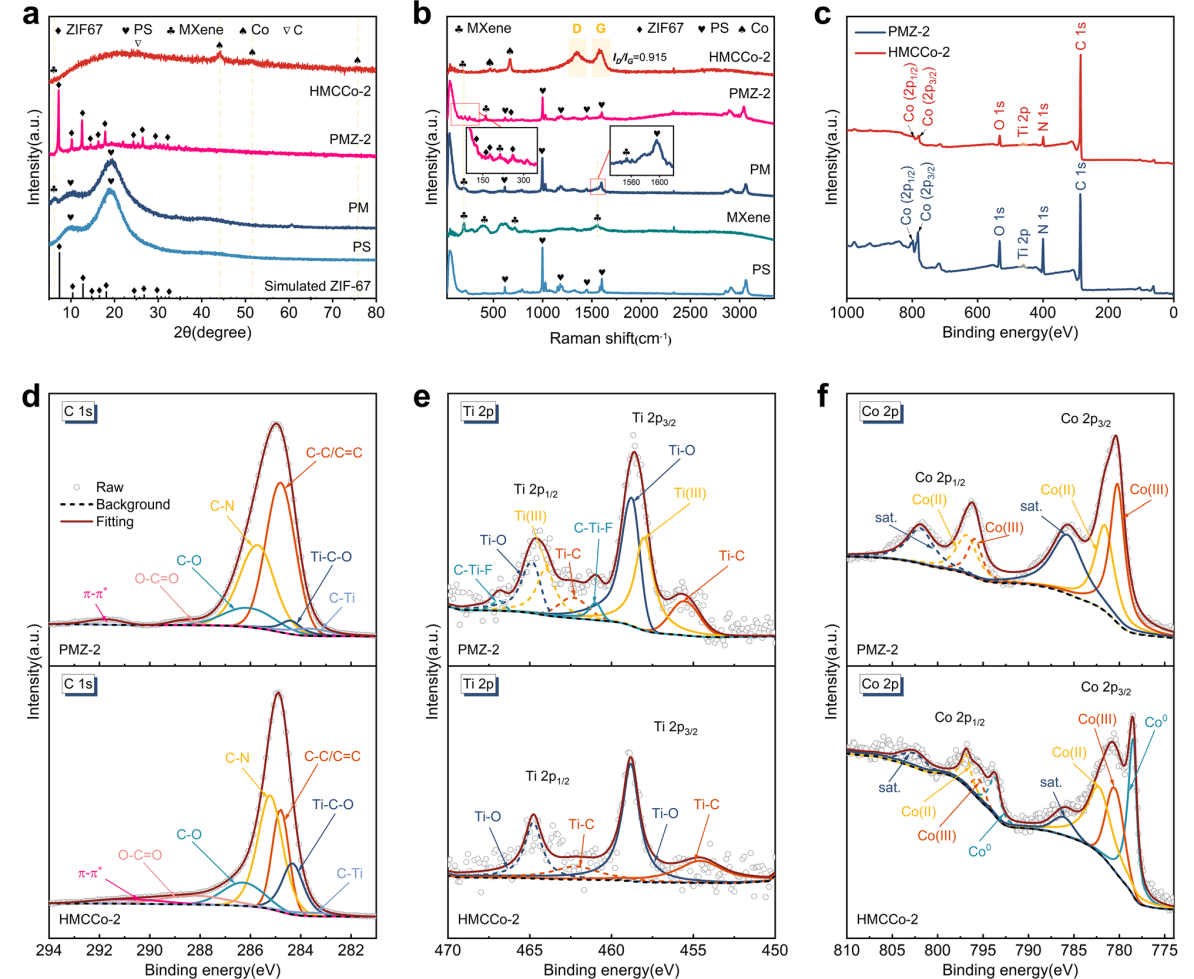
**a** XRD patterns for ZIF67, PS, PM, PMZ-2, and HMCCo-2. **b** Raman spectra of PS, MXene, PM, PMZ-2, and HMCCo-2. **c** XPS survey. **d** C 1*s* spectra of PMZ-2 and HMCCo-2. **e** Ti 2*p* spectra of PMZ-2 and HMCCo-2. **f** Co 2*p* spectra of PMZ-2 and HMCCo-2

Figure 3a shows the XRD pattern for the prepared absorbent material. HMCCo-2 had a small, broad peak at 25.6°, which was believed to originate from the (002) crystal plane of the hexagonal graphite CNTs [66]. The peaks at 44.2°, 51.6°, and 75.8° represented the (111), (200), and (220) planes of metallic cobalt with the face-centered cubic structure (JCPDS No. 15–0806), respectively. The small peak for the PM at 6.2° arose from the (002) plane of MXene. Combined with the SEM images, it was found that MXene was successfully encapsulated onto the PS spheres. However, other smaller peaks for the (004), (006), (008), and (0010) planes of MXene shown in Fig. S4 were not observed for PM [67]. The peaks at 7.2°, 10.2°, 12.58°, 14.58°, 16.3°, 17.86°, 24.34°, 26.46°, 29.46°, and 32.2° for PMZ-2 were due to the (002), (112), (022), (013), (222), (233), (134), (044), and (235) planes of ZIF67, respectively [68]. The disappearance of these peaks and generation of the cobalt peaks indicated the conversion of the Co^2+^ ions in ZIF67 into metallic Co particles.

The graphite properties shown via XRD were confirmed with Raman spectroscopy (Fig. 3b). The D band (1355.4 cm^−1^) and G band (1589.1 cm^−1^) caused by *sp*^2^ hybridization were observed in HMCCo-2. The heights of the D band and G band represent the contents of disordered carbon and graphite carbon, respectively, in the lattice structure [69]. The ratio of ${I}_{D}$ and ${I}_{G}$ in the figure was 0.915, and the strong D band indicated that there may be defects in the graphite carbon or MXene nanosheets in the sample. The main Raman peaks for the PS spheres were at 621, 1030, 1177, 1451, and 1603 cm^−1^ [70]. The characteristic Raman peaks for PS in PMZ-2 disappeared in HMCCo, implying the removal of PS microspheres, which is consistent with the results of thermogravimetric analysis (Fig. S5). The peaks at 201 and 723 cm^−1^ arose from Ti–C and C–C vibrations, while the peak at 620 cm^−1^ was generated by vibrations of the –OH groups. In addition, a characteristic peak for MXene appeared at 1553 cm^−1^, which was also shown in PM [71, 72]. The peaks at 128, 162, 179, 260, and 687 cm^−1^ for PMZ-2 were the characteristic peaks of ZIF67, where those at 162 and 179 cm^−1^ were generated by N–Co–N vibrations, and the peaks at 260 and 687 cm^−1^ were derived from the C–CH_3_ bonds in imidazole. The appearance of these peaks indicated the successful growth of ZIF67 on the surface of PM. The characteristic peak for the MXene in HMCCo-2 shifted to 191 cm^−1^, indicating that the interlayer spacing of MXene had increased after calcination. The peaks at 472 and 675 cm^−1^ represented the Raman active E_g_ and A_1g_ modes for CoO_*x*_ [73]. Therefore, there was oxidized cobalt in the product, but it was not detected by XRD, indicating that the Co nanoparticles in the sample may be encapsulated by thin layers of cobalt oxide. No metal Co particle vibration was observed in Raman, which was consistent with experimental results reported in the literature [74, 75].

The chemical compositions and valence states of the PMZ-2 and HMCCo-2 samples were characterized with XPS. Figure 3c–f shows that the sample was composed of C, Ti, Co, N, O, and F. The C 1*s* peaks at 283.4, 284.3, 284.8, 285.3, 286.2, and 288.2 eV were from C–Ti, Ti–C–O, C–C/C=C, C–N, C–O, and O–C=O groups, respectively [15] (Fig. 3d). The Ti 2*p*_3/2_ peaks at 455.4, 457.9, 458.8, and 460.9 eV for the PMZ sample before heat treatment shown in Fig. 3e corresponded to Ti-C, Ti(III), Ti–O, and C–Ti–F groups, respectively. The corresponding Ti 2*p*_1/2_ peaks were at 462.3, 464, 464.8, and 466.9 eV, respectively. The HMCCo-2 obtained after calcination only had four distinct peaks, namely, those for Ti–C 2*p*_3/2_ and Ti–C 2*p*_1/2_ at 454.8 and 462.2 eV, as well as two peaks for Ti–O [76]. Due to the absence of TiO_2_ peaks in the XRD and Raman data, it is believed that the terminal groups of MXene in the heat-treated sample were replaced by Ti–O [77]. After calcination, metallic Co (Co°) and Co compounds were present in the sample. As shown in Fig. 3f, the peak positions at 778.5 and 793.6 eV correspond to the 2*p*_3/2_ and 2*p*_1/2_ energy levels of metallic Co, respectively, and those at 780.6 and 795.6 eV were the 2*p*_3/2_ and 2*p*_1/2_ binding energies of Co(III), respectively. The peaks at 782.3 and 796.9 eV corresponded to the 2*p*_3/2_ and 2*p*_1/2_ states of Co(II), and the satellite peaks were at 786.3 and 802.4 eV [75, 78]. There was no metallic Co present in the untreated PMZ-2 sample. The deconvoluted N 1* s* peaks (Fig. S6a) showed the presence of pyridinic N (398.9 eV), graphitic N (400.8 eV), and oxidized N (403.5 eV) in the sample. According to prior research, abundant pyridinic Ns in HMCCo-2 improve the conductivity [79]. There were five binding energies in the O 1*s* (Fig. S6b) spectrum of the HMCCo-2 sample for Ti–O (530.3 eV), C–Ti–O_*x*_ (531.7 eV), Ti–C–(OH)_*x*_ (532.7 eV), H_2_O (533.7 eV) and O–F (535.4 eV) [80]. The peaks for Ti–C–(OH)_*x*_ and O–F appeared before calcination. The F 1*s* (Fig. S6c) peaks for the C–Ti–F (684.4 eV) groups appeared in the samples before and after calcination [81].

### Electromagnetic Wave Absorption Performance of HMCCo

**Fig. 4:**
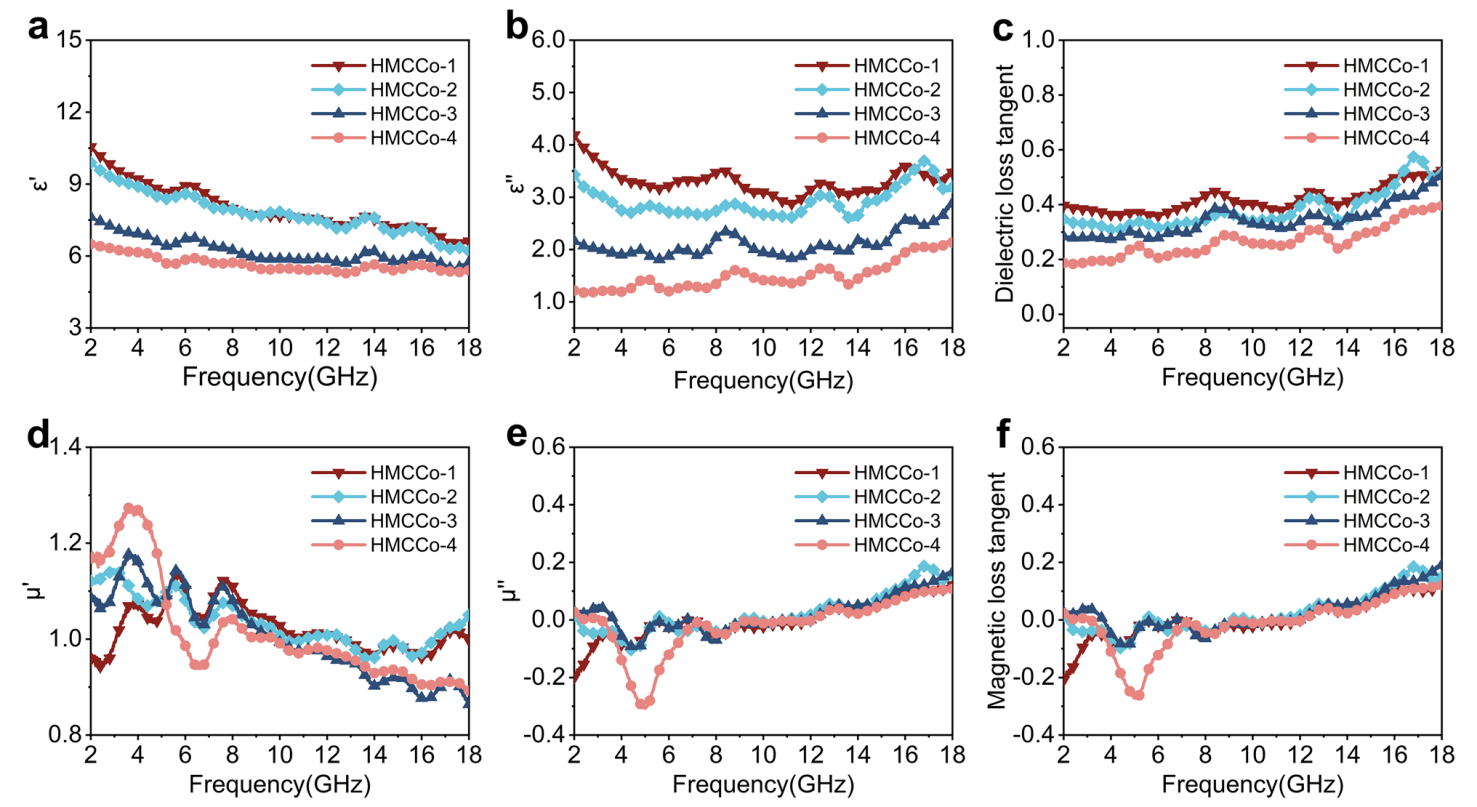
**a** Real and **b** imaginary parts of the complex permittivity, **c** dielectric loss tangent values, **d** real and **e** imaginary parts of the permeability, and **f** magnetic loss tangent values for HMCCo-1, HMCCo-2, HMCCo-3, and HMCCo-4

The absorption of an absorber is closely related to its complex permittivity (${\varepsilon }_{r}={\varepsilon }{\prime}-{j\varepsilon }^{{\prime}{\prime}}$) and complex permeability (${\mu }_{r}={\mu }{\prime}-{j\mu }^{{\prime}{\prime}}$). Generally, the real components (${\varepsilon }^{ {\prime}}$ and ${\mu }{\prime}$) and imaginary components (${\varepsilon }^{{\prime}{\prime}}$ and ${\mu }^{{\prime}{\prime}}$) represent the storage and dissipation capacities of the absorber with electromagnetic waves, respectively [76]. To investigate the effect of C–Co content on the performance of the absorber, the electromagnetic parameters were tested within the frequency range 2–18 GHz for all paraffin sample coaxial rings with 15 wt% filler ratios. As shown in Fig. 4, as the coverage of C–Co increased, ${\varepsilon }{\prime}$ and ${\varepsilon }^{{\prime}{\prime}}$, the relative complex dielectric constant and dielectric loss tangent (${\text{tan}}{\delta }_{\upvarepsilon }={\varepsilon }^{{\prime}{\prime}}/{\varepsilon }{\prime}$), showed decreasing trends. This indicated that the increased relative content of magnetic C–Co weakened the dielectric storage and dissipation capabilities of the absorber. The free electron theory indicates that a high ${\varepsilon }^{{\prime}{\prime}}$ leads to high conductivity, and with higher conductivities, more electromagnetic waves are reflected, which hinders the entry of electromagnetic waves into the interior of the absorber [82]. The real and imaginary parts decreased with increase in C–Co content because anchoring of the magnetic C–Co networks weakened the electronic transitions between the conductive networks constructed of pure MXene. The decreasing trend for the real part with increase in frequency was consistent with the typical frequency dispersion effect. In addition, ${\varepsilon }^{{\prime}{\prime}}$ and ${\text{tan}}{\delta }_{{\text{e}}}$ exhibited some fluctuations in the range 2–18 GHz, which were due to relaxation peaks caused by polarization effects. The Cole–Cole diagram (Fig. S6) based on Debye theory demonstrates the polarization behavior of preparing absorbers, and the specific formula as follows [83, 84]:

3 $${\left({\varepsilon }{\prime}-\frac{{\varepsilon }_{s}+{\varepsilon }_{\infty }}{2}\right)}^{2}+{\left({\varepsilon }^{{\prime}{\prime}}\right)}^{2}={\left(\frac{{\varepsilon }_{s}-{\varepsilon }_{\infty }}{2}\right)}^{2}$$

(3where ${\varepsilon }_{s}$ and ${\varepsilon }_{\infty }$ are the static dielectric constant and relative dielectric constant at the high-frequency limit, respectively. In the Cole–Cole diagrams of the HMCCo samples, some irregular semicircles were observed. This indicated that polarization relaxation occurred in the prepared sample due to the influence of an external electromagnetic field. The main stimulating factors for relaxation are interface polarization and dipole planning [85, 86]. In the HMCCo series, interfacial polarization was attributed to the accumulation of positive and negative charges from the C–Co/MWCNTs, MWCNTs/MXene, and Co nanoparticles/MWCNTs. Dipolar polarization was mainly caused by abundant defects in the MWCNTs and MXene and polar functional groups with different charges. Therefore, the constructed hollow multilayer MXene/MWCNTs/C–Co spherical shell structure contributed to dielectric loss.

Magnetic losses caused by magnetic metal cobalt nanoparticles are important for enhancing electromagnetic wave dissipation. As shown in Fig. 4d–f, there were several resonance peaks for the complex permeability of the absorber, and within the range 2–18 GHz, the values of ${\mu }{\prime}$ and ${\mu }^{{\prime}{\prime}}$ were relatively stable. The negative fluctuation at ~ 5 GHz displayed in the imaginary part of the HMCCo-4 spectrum may have been caused by the direct current generated via migration of charge carriers inside the material due to the action of an external magnetic field [87]. Additionally, the magnetic loss tangent (${\text{tan}}{\delta }_{\upmu }={\mu }^{{\prime}{\prime}}/{\mu }{\prime}$) values of the four samples were all within the range − 0.3 to 0.2 and showed small upward trends. It is worth noting that in the range 2–18 GHz, ${\text{tan}}{\delta }_{\upvarepsilon }$ was larger than ${\text{tan}}{\delta }_{\upmu }$, which demonstrated that the main form of electromagnetic wave consumption in the prepared materials was dielectric loss. Further magnetic loss mechanisms were indicated by the ${\mu }^{{\prime}{\prime}}{\left({\mu }{\prime}\right)}^{-2}{f}^{-1}$-frequency curves [88]. The curve fluctuation shown in Fig. S8 indicated that eddy current losses can be ignored. Due to the confinement effect and microscale of the Co particles, natural resonance and exchange resonance were the main causes of magnetic loss in the prepared composite materials. The magnetic parameters shown in Fig. S9 for the samples before and after calcination were measured at room temperature with a vibrating sample magnetometer. Typical magnetism is shown in Fig. S9b, and the saturation magnetization (*M*_s_) of HMCCo-2 was approximately 27.93 emu g^−1^. Considerable strength was contributed by the cobalt metal, and in addition, the sample exhibited low coercivity (*H*_c_ = 140.3 G) and remanence (*M*_r_ = 9.9 emu g^−1^).

**Fig. 5:**
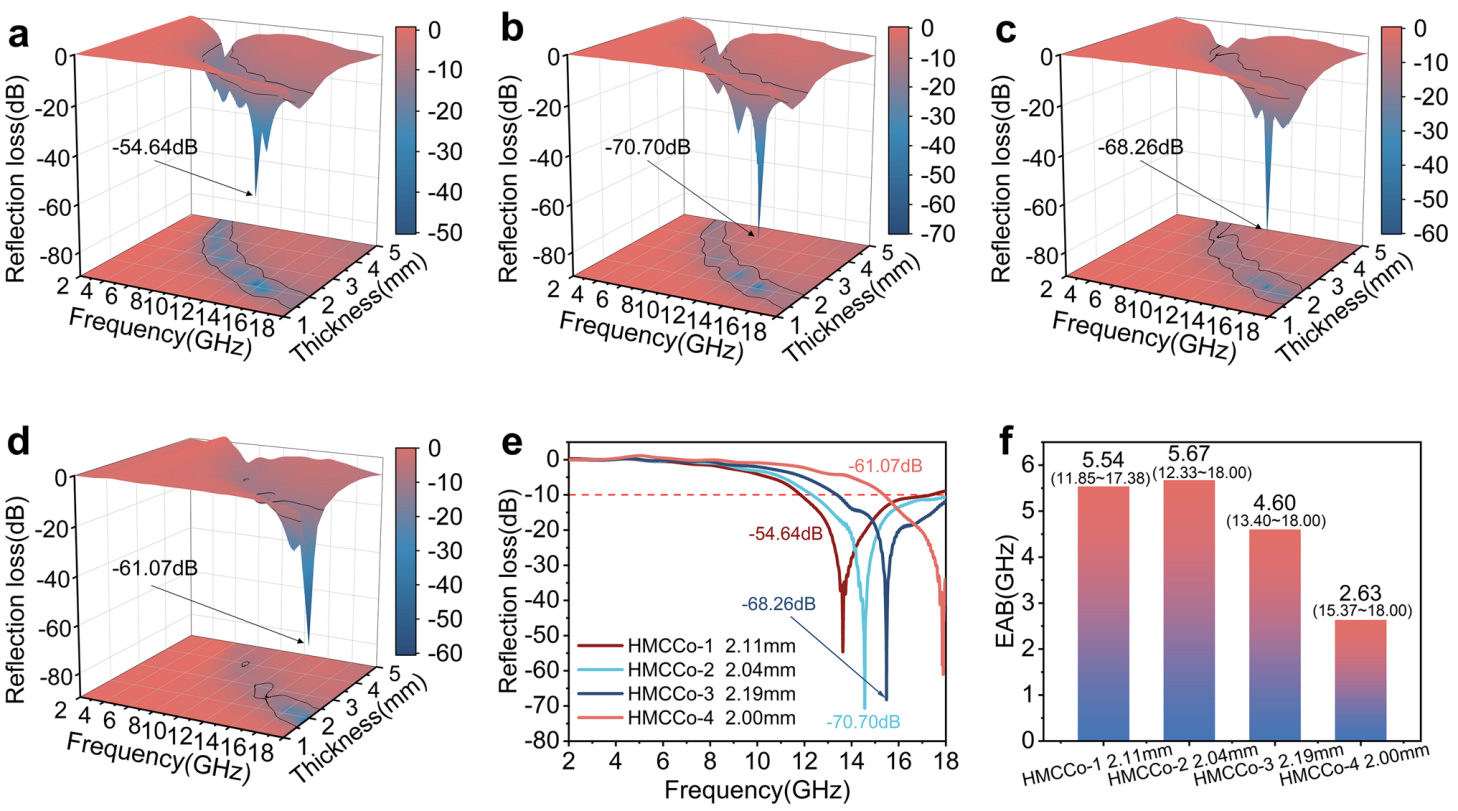
3D reflection losses of **a** HMCCo-1, **b** HMCCo-2, **c** HMCCo-3, and **d** HMCCo-4. **e** Reflection losses with matching thicknesses of the HMCCo samples. **f** EABs of the HMCCo series

The absorption capacities of samples with thicknesses of 1–5 mm are demonstrated with 3D images (Fig. 5). Figure 5a–f shows the outstanding absorption performance of the prepared material. The RL values were calculated with Eqs. 4 and 5. The four samples reached RL_min_ values of 13.63, 14.56, 15.49, and 17.89 GHz, which were − 54.64, − 70.70, − 68.26, and − 61.07 dB, respectively. Generally, within the frequency range corresponding to RL values less than − 10 dB, 90% of electromagnetic waves can be absorbed by the absorber, and this frequency range is called the effective absorption bandwidth. The effective absorption bandwidth (EAB) values of the HMCCo series with matching thicknesses were 5.54, 5.67, 4.60, and 2.63 GHz, respectively, as shown in Fig. 5e. All samples reached the RL_min_ value at ~ 2 mm, and the performance of HMCCo-2 was superior to those of the other samples. The optimal RL was − 70.70 dB at 14.56 GHz, with an EAB of 5.67 GHz (12.33 ~ 18 GHz). A more detailed RL analysis of the HMCCo series at 2–18 GHz is shown in Fig. S10a. As the thickness of the absorber increased, the optimal RL peak shifted toward low frequencies, which is consistent with the well-known 1/4 wavelength matching model [89].

Typically, the mechanisms by which materials enhance electromagnetic wave absorption can be understood by discussing their ability to capture and attenuate incident waves. Good impedance matching ensures that as many electromagnetic waves as possible enter the interior of the absorber. This is evaluated with the normalized characteristic impedance ($Z$), which is calculated according to Eq. 4:

4 $$Z=\left|{Z}_{{\text{in}}}/{Z}_{0}\right|=\sqrt{{\mu }_{r}/{\varepsilon }_{r}}{\text{tanh}}\left(j\frac{2\pi fd}{c}\sqrt{{\mu }_{r}{\varepsilon }_{r}}\right)$$

Z values closer to 1 indicate better impedance matching [90, 91]. As shown in Fig. S10b, compared to the other two samples, HMCCo-1 and HMCCo-2 had larger optimum $\left|{Z}_{{\text{in}}}/{Z}_{0}\right|$ values (near the wathet area), which enabled the electromagnetic waves to enter the interior of the material. The curve for the $Z$ value in Fig. S11b indicated that the area of the curve for the HMCCo-2 sample was larger in the area near $Z$=1 than those of the other three samples. HMCCo-4 showed the worst impedance match. This indicated that impedance matching first increased and then decreased with increase in C–Co. Note that the impedance of the composite material was related to the electromagnetic parameters of the material, and there was an optimal impedance value in a certain region for each sample, which means that the constructed multilayer hollow spherical structure optimized the electromagnetic parameters of the composite system. The presence of metallic Co and the carbon nanotubes regulated the magnetic permeability and dielectric constant of the composite system. Furthermore, the attenuation constant $\alpha$ was used to determine the overall efficiency of the absorber in consuming electromagnetic waves. The $\alpha$ value is represented by Eq. 5 [92, 93]:

5 $$\alpha =\sqrt{2}\pi f/c\sqrt{\left({\mu }^{{\prime}{\prime}}{\varepsilon }^{{\prime}{\prime}}-{\mu }{\prime}{\varepsilon }{\prime}\right)+\sqrt{{\left({\mu }^{{\prime}{\prime}}{\varepsilon }^{{\prime}{\prime}}-{\mu }{\prime}{\varepsilon }{\prime}\right)}^{2}+{\left({\mu }{\prime}{\varepsilon }^{{\prime}{\prime}}+{\mu }^{{\prime}{\prime}}{\varepsilon }{\prime}\right)}^{2}}}$$

The frequency-dependent $\alpha$ curve (Fig. S11a) shows that within 2–18 GHz, the ability of all absorbers to attenuate electromagnetic waves increased with increase in frequency. In the high-frequency range, attenuation by the absorber first increased and then decreased. The HMCCo-2 sample exhibited strong attenuation at high frequencies, and when the $\left|{Z}_{{\text{in}}}/{Z}_{0}\right|$ value approached 1, RL reached its extreme value of − 70.70 dB, and the matching thickness was 2.04 mm, as shown in Fig. S12. In contrast, an appropriate C–Co loading provided a better balance between impedance matching and the attenuation constant, thereby achieving efficient absorption of electromagnetic waves. EAB is one of the indicators used for evaluating the performance of absorbers, as shown in Fig. S10c. Thickness of 1.5–5 mm for the HMCCo series resulted in an EAB of 13.1 GHz (4.9–18 GHz) for HMCCo-2, which was slightly narrower than the EAB of HMCCo-1 (4.75–18 GHz), but better than those of the other samples. Both HMCCo-1 and HMCCo-2 reached their widest EAB near 2 mm (Fig. S11c), which subsequently narrowed due to the increased thicknesses, while HMCCo-3 and HMCCo-4 achieved their largest EAB near 3 mm. This means that increasing the amount of C–Co to obtain a wider EAB requires increasing the thickness of the absorber.

To explore the advantages of ZIF67 and MXene synergy, four groups of comparative experiments were carried out. As shown in Fig. S13a, pure ZIF67 maintained a sharp dodecahedral structure after calcination at 600 °C, and no CNTs were generated. The growth of ZIF67 on the PS microspheres did not uniformly and completely envelop the entire PS spheres (Fig. S13b1). PS@ZIF67 After calcination, the template was removed, the ZIF67 particles aggregated, and some of the calcined ZIF67 grew CNTs (Fig. S13b2). MXene provided binding sites for the ZIF67 particles, so the MXene surface was more uniformly wrapped by ZIF67, as shown in Fig. S13c1. In the calcined MXene@ZIF67 sample, the originally grown ZIF67 particles between the layers were completely replaced by crisscrossing CNTs (Fig. S13c2). It is worth noting that the growth directions of the CNTs at this time were random. Figure S12d shows that the PS@MXene spheres without ZIF67 wrapped in the outer layer shrank after calcination, making it impossible to maintain the full spherical shell structure. The RL values of the comparison group are shown in Fig. S13, and all were inferior to that of the experimental group. The reason is that the attenuation constant and impedance matching of the comparison group did not balance each other (as shown in Fig. S15). In addition, the corresponding electromagnetic parameters are shown in Fig. S14. In summary, the structure designed in this paper made full use of the rich functional groups on the surface of MXene to provide binding sites for the growth of ZIF67. Additionally, the C–Co obtained after calcining ZIF67 was used as the exoskeleton supporting the MXene spherical shells, thus ensuring a full spherical shell structure. In addition, the PS served as an abundant carbon source and provided the basic conditions for in situ growth of the MWCNTs with spherical orientations. Perfect coordination of the structure and components resulted in an excellent performance by the composite absorbing material.

In summary, sample HMCCo-2 exhibited the best morphology and most complete structure. Additionally, considering the attenuation and impedance matching of the sample, sample HMCCo-2 exhibited the minimum RL and the widest EAB with a small matching thickness. Therefore, it is believed that the ratio of sample HMCCo-2 could enhance electromagnetic wave absorption. Moreover, in comparing the electromagnetic properties of the PMZ-2 sample (Fig. S16) without calcination, the C–Co layer and MWCNTs formed after calcination optimized impedance matching of the composite material and significantly improved the absorption and consumption of electromagnetic waves.

### Effect of Calcination Temperature on the Properties of HMCCo

According to some studies, the temperature affects the degree of carbonization of ZIF67, which may cause changes in the electromagnetic properties of the HMCCo series [94, 95]. Therefore, based on the optimal the MXene to ZIF67 ratio, the influence of temperature on the sample morphology and absorption performance was explored by adjusting the calcination temperature during CVD. Specifically, the other conditions used for the tube furnace remained unchanged, including the nitrogen flow rate, the temperature rise and fall rates, and the insulation duration. Only the insulation temperature was changed, and five different temperature parameters were set from 400 to 800 °C for the experiments. The electromagnetic parameters of samples measured at different calcination temperatures with the vector network analyzer are shown in Fig. S17. The ${\varepsilon }{\prime}$ of the samples obtained at 400 and 500 °C was approximately 2, ${\varepsilon }^{{\prime}{\prime}}$ was almost 0, and ${\text{tan}}{\delta }_{\upvarepsilon }$ also fluctuated slightly at approximately 0. This indicated that a heat treatment temperature below 500 °C was insufficient to improve the dielectric properties of the composite samples. Additionally, ${\mu }^{{\prime}{\prime}}$ and ${\text{tan}}{\delta }_{\upmu }$ were almost zero, indicating that the samples obtained at these two sets of temperatures had no magnetic losses. When the temperature exceeded 600 °C, the electromagnetic performance of the sample was significantly improved. It was observed that ${\varepsilon }{\prime}$ showed an upward trend as the calcination temperature increased. As the temperature increased, ${\varepsilon }^{{\prime}{\prime}}$ exhibited more significant fluctuations, indicating that phenomena such as interface polarization and dipole polarization within the material were more prevalent. In addition, ${\mu }{\prime}$ first increased and then decreased with increase in temperature, and the sample obtained at 700 °C had the highest ${\mu }{\prime}$. The resonance behavior of the samples calcined at 700 and 800 °C was stronger, and the negative peaks were more prominent, which was attributed to the increased direct current generated by the movement of charge carriers inside the material. Overall, ${\text{tan}}{\delta }_{\upvarepsilon }$ was higher than ${\text{tan}}{\delta }_{\upmu }$, indicating that the material consumed electromagnetic waves mainly due to dielectric loss.

Figure S18 shows that samples with temperatures above 600 °C exhibited significant and complex relaxation behavior, and the influence of eddy current loss was reduced. More magnetic losses came from natural resonance and exchange resonance. The resonance peak was more pronounced in samples treated at 800 °C, and they showed the strongest attenuation ability. However, strong attenuation does not necessarily mean strong RL performance. As mentioned earlier, the evaluation of RL requires comprehensive attenuation and impedance matching. From the $Z$ values shown in Fig. S19, it can be seen that the values of approximately 1 for the $Z$ values of the samples obtained from carbonization at 600 and 700 °C were significantly wider than those of the samples obtained at 800 °C. This may be due to the high calcination temperatures, increased carbonization degree of the sample, and enhanced dielectric properties, resulting in more electromagnetic waves being reflected by the sample, thereby weakening impedance matching. This conjecture can be verified with the Raman spectrum (Fig. S20). As the calcination temperature increased, the peak intensities for Co gradually increased compared to those of the *D* and *G* bands, indicating that the cobalt in the outermost layer of ZIF67 was largely metallic cobalt. In addition, the value of ${I}_{D}/{I}_{G}$ increased with increase in temperature, indicating more defects in the MXene or carbon nanotubes within the material, which may be the reason for more relaxation peaks in ${\varepsilon }^{{\prime}{\prime}}$. However, no significant Co peaks were observed after calcination at 400 and 500 °C, indicating that ZIF67 maintained its original morphology at lower temperatures.

**Fig. 6:**
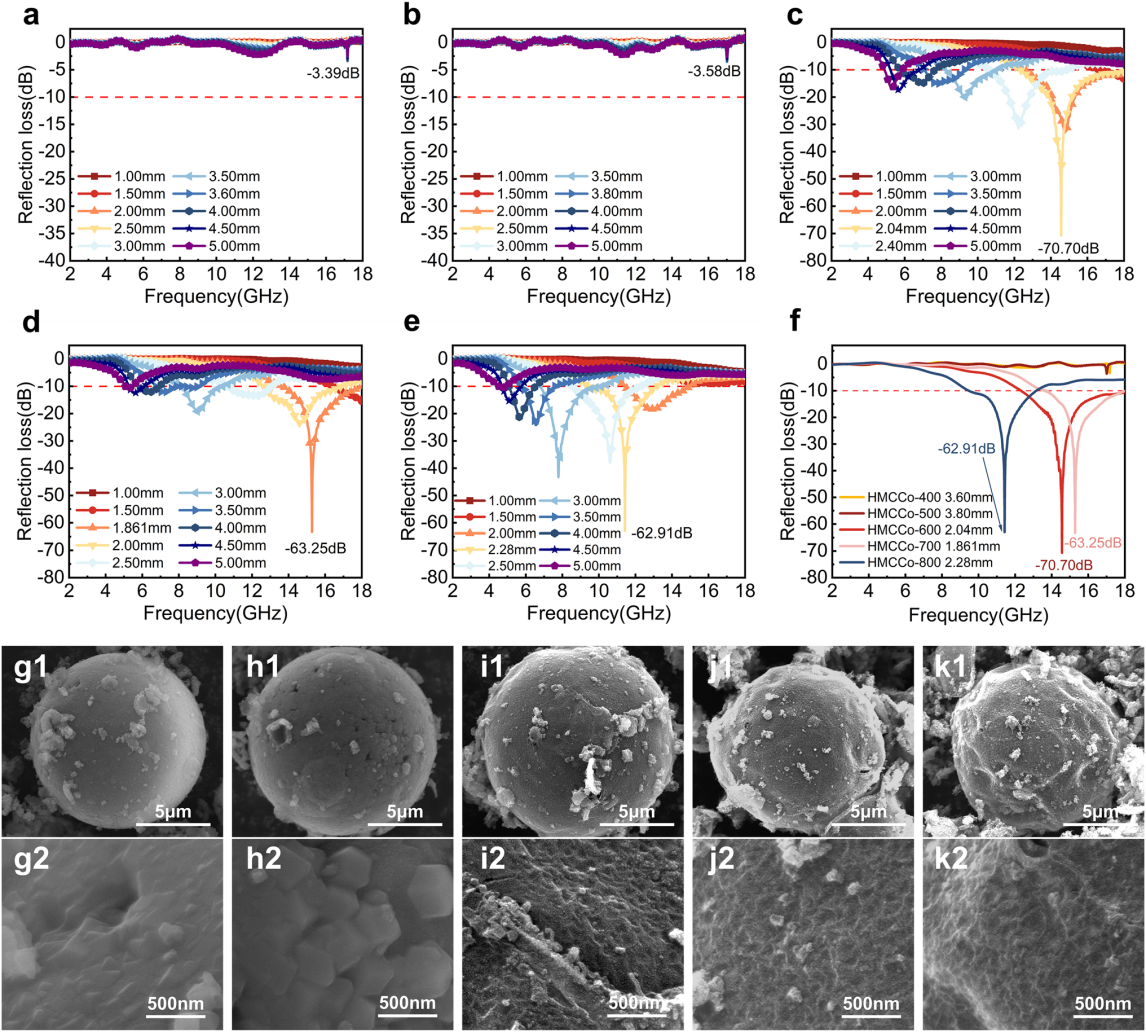
RL plots for **a** HMCCo-400, **b** HMCCo-500, **c** HMCCo-600, **d** HMCCo-700, and **e** HMCCo-800 at thicknesses of 1 to 5 mm. **f** RL curves for samples prepared at different calcination temperatures with matched thicknesses. SEM images of **g1** HMCCo-400, **h1** HMCCo-500, **i1** HMCCo-600, **j1** HMCCo-700, and **k1** HMCCo-800. SEM detail images of **g2** HMCCo-400, **h2** HMCCo-500, **i2** HMCCo-600, **j2** HMCCo-700, and **k2** HMCCo-800

In general, the compositions and morphologies of the products were indeed affected by the carbonization temperatures, thereby altering electromagnetic wave absorption by the samples. Based on the electromagnetic parameters obtained from testing, the RL values of the absorber were calculated for thicknesses from 1 to 5 mm, as shown in Fig. 6. Obviously, 400 and 500 °C were not sufficient to carbonize ZIF67, and in SEM images, the surfaces of these samples maintained the polyhedral morphology of ZIF67. As the temperature was increased, the minimum RL values of the prepared samples gradually decreased, ranging from − 70.70 dB at 14.56 GHz, − 63.25 dB at 15.28 GHz to − 62.91 dB at 11.42 GHz, respectively. Notably, the sample calcined at 700 °C exhibited the optimal RL with the thinnest matching thickness, with a width of only 1.861 mm. Moreover, the electromagnetic parameters were closely related to the chemical composition and structure of the sample. The SEM microscopic morphology diagram in Fig. 6g–k shows that as the temperature increased, while the PS template was removed, the spherical shell underwent slight shrinkage and indentation at high temperatures. This means that ZIF67 was heavily carbonized and did not serve as the exoskeleton. Therefore, the consumption of electromagnetic waves was restricted to some extent. The EAB was also affected by the temperature. The EAB of the sample calcined at 700 °C decreased to 4.38 GHz (13.62–18 GHz), while at 800 °C, it decreased to 3.70 GHz (9.46–13.16 GHz), and the effective absorption range was shifted to a lower frequency range at this temperature. As shown in Fig. S21, the optimal EAB was obtained by adjusting the thickness of the sample calcined at 600 °C from 1.5 to 5 mm.

### Mechanism for Electromagnetic Wave Absorption Loss

The hollow spherical shell structure reduced the density of the material, improved the electromagnetic parameters and impedance matching in the sample [96, 97]. Single-component materials are prone to impedance mismatch and lack of conductive networks, so other components are often introduced for regulation. Multicomponent heterogeneous interfaces exhibit capacitance-like modes between them, leading to polarization relaxation behavior of space charges under alternating electric fields, which results in polarization loss [98, 100, 101]. In addition, the process by which the components intervene leads to defects, phase transitions, and other major factors inducing polarization in the material [102, 103]. In this study, the above key factors affecting the MA performance were considered comprehensively, and the electromagnetic parameters were adjusted to a suitable range through the construction of MXene-based hollow core–shell structure and the introduction of magnetoelectric synergism, which ensured that the composites exhibited the best electromagnetic wave attenuation performance.

**Fig. 7:**
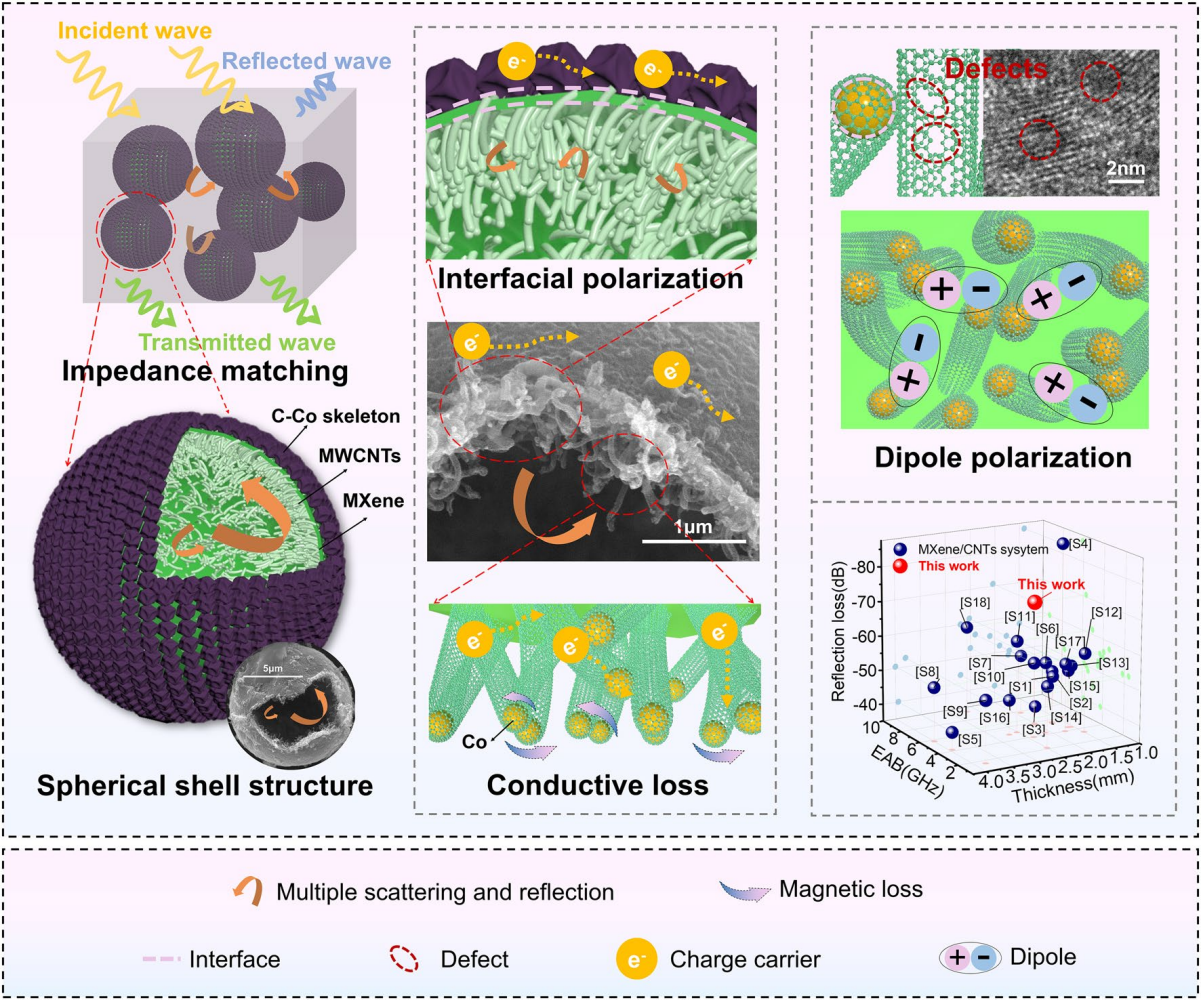
Mechanism for electromagnetic wave loss

The mechanism for electromagnetic wave consumption by the HMCCo absorber is shown in Fig. 7. First, based on the good impedance match of the material, most electromagnetic microwaves can enter the absorber, which is also a prerequisite for consuming electromagnetic waves. Meanwhile, a few microwaves are reflected into the air, and even fewer microwaves are transmitted. Second, the interesting hollow layered spherical shells provided multiple scattering and reflection of the incident electromagnetic waves inside the spherical shell. The cavities optimized the MA properties of the material more than solid structures that also had hierarchical structures [104]. Additionally, the unique inner network of MWCNTs also extended the paths for multiple scattering of the electromagnetic waves and increased the ability of the prepared material to consume waves. Furthermore, the carbon nanotubes were interweaved with each other to form a three-dimensional conductive network, which, along with the C–Co skeleton layer, facilitated migration and jumping of charge carriers, which converted the electromagnetic wave energy into thermal energy and dissipated it. As mentioned earlier, dielectric loss is the main mechanism for consumption of electromagnetic waves, and dielectric loss includes interface polarization and dipole polarization. The alternating layer structure of MXene sandwiched between the MWCNT layer and the C–Co skeleton layer provided a rich heterogeneous interface, where charges of the different electrical properties accumulated and formed a structure similar to that of a micro-capacitor and enhanced interface polarization. In addition, numerous dipoles were formed between the many defects and different electrical functional groups in the material, which aided dielectric loss. Finally, because the prepared material contained metallic cobalt, the natural resonance of the cobalt nanoparticles and the exchange resonance between different cobalt particles led to magnetic loss. In summary, good impedance matching, multiple composite structure design, and the synergistic effect of multiple loss mechanisms provided the HMCCo absorbers with excellent electromagnetic wave absorption. As shown in the small image in Fig. 7, the absorption by HMCCo-2 was highly competitive with those of most reported MXene/CNT absorption materials. More detailed comparative information is shown in Table S2.

## Conclusions

A novel structure with MWCNTs growing toward the interiors of spherical shells was constructed via self-assembly and high-temperature calcination. The as-prepared hollow MXene sphere weaved MWCNTs with a C–Co skeleton can enhance the multiple losses and polarization effects simultaneously. The optimal sample exhibits a minimum RL of − 70.70 dB at 14.56 GHz with a matching thickness of only 2.04 mm. Meanwhile, by adjusting the thickness from 1.5 to 5 mm, a broad EAB of 13.1 GHz was obtained. In addition, the effects of temperature on the morphology and electromagnetic properties of the ZIF67 composite material were also studied. This research showed that the appropriate temperature safeguards both the growth of MWCNTs and the maintenance of a hollow core–shell structure that facilitates microwave absorption. Therefore, the unique structural design of this study provides a reference for the design of efficient electromagnetic wave-absorbing materials in the future.

## Supplementary Information

Below is the link to the electronic supplementary material.

Supplementary file1 (PDF 3478 KB)
