# Supplementary material for: MXene Hollow Spheres Supported by a C–Co Exoskeleton Grow MWCNTs for Efficient Microwave Absorption
Source: Nanomicro Lett. 2024 Feb 2;16:107. doi: 10.1007/s40820-024-01326-3 (PMC10837412; doi:10.1007/s40820-024-01326-3)
Supplement: Supplementary file 1 — Supplementary file1 (PDF 3478 KB) [file 40820_2024_1326_MOESM1_ESM.pdf]

Supporting Information for

MXene Hollow Spheres Supported by a C-Co Exoskeleton Grow MWCNTs for Efficient Microwave Absorption

Ze Wu[1], Xiuli Tan[1], Jianqiao Wang[1], Youqiang Xing[1], Peng Huang[1], Bingjue Li[1], Lei Liu[1,]*

[1]School of Mechanical Engineering, Southeast University, Nanjing 211189, P. R. China

*Corresponding author. E-mail: liulei@seu.edu.cn (Lei Liu)

Supplementary Figures and Tables

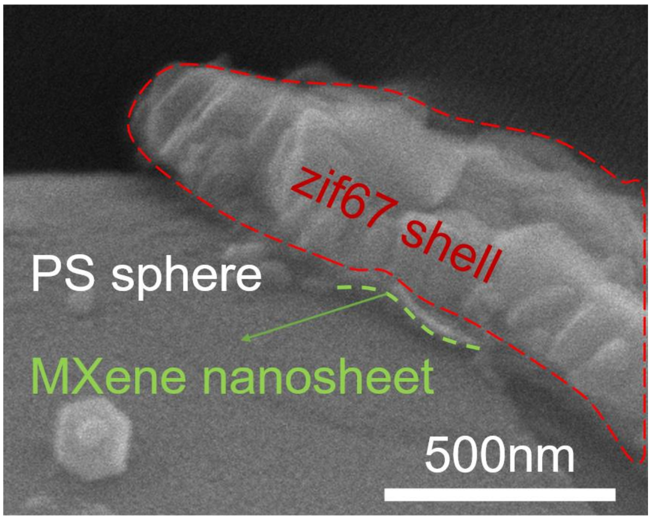

Fig. S1 SEM image of PS@MXene@ZIF67 with incomplete MXene coating

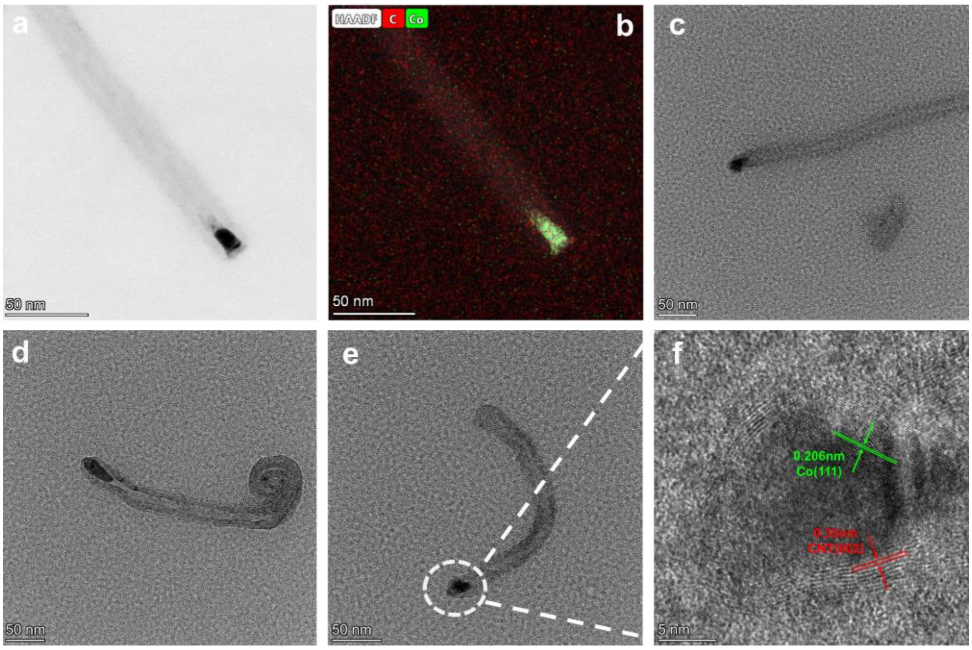

Fig. S2 Disperse HMCCo-2 in ethanol solution and sonicate for 15 minutes before taking TEM images. **a** Single carbon nanotube separated after ultrasound. **b** Elements mapping for single carbon nanotubes. **c-e** Single carbon nanotubes separated after ultrasound. **f** High resolution TEM images of carbon nanotube tips and Co nanoparticle

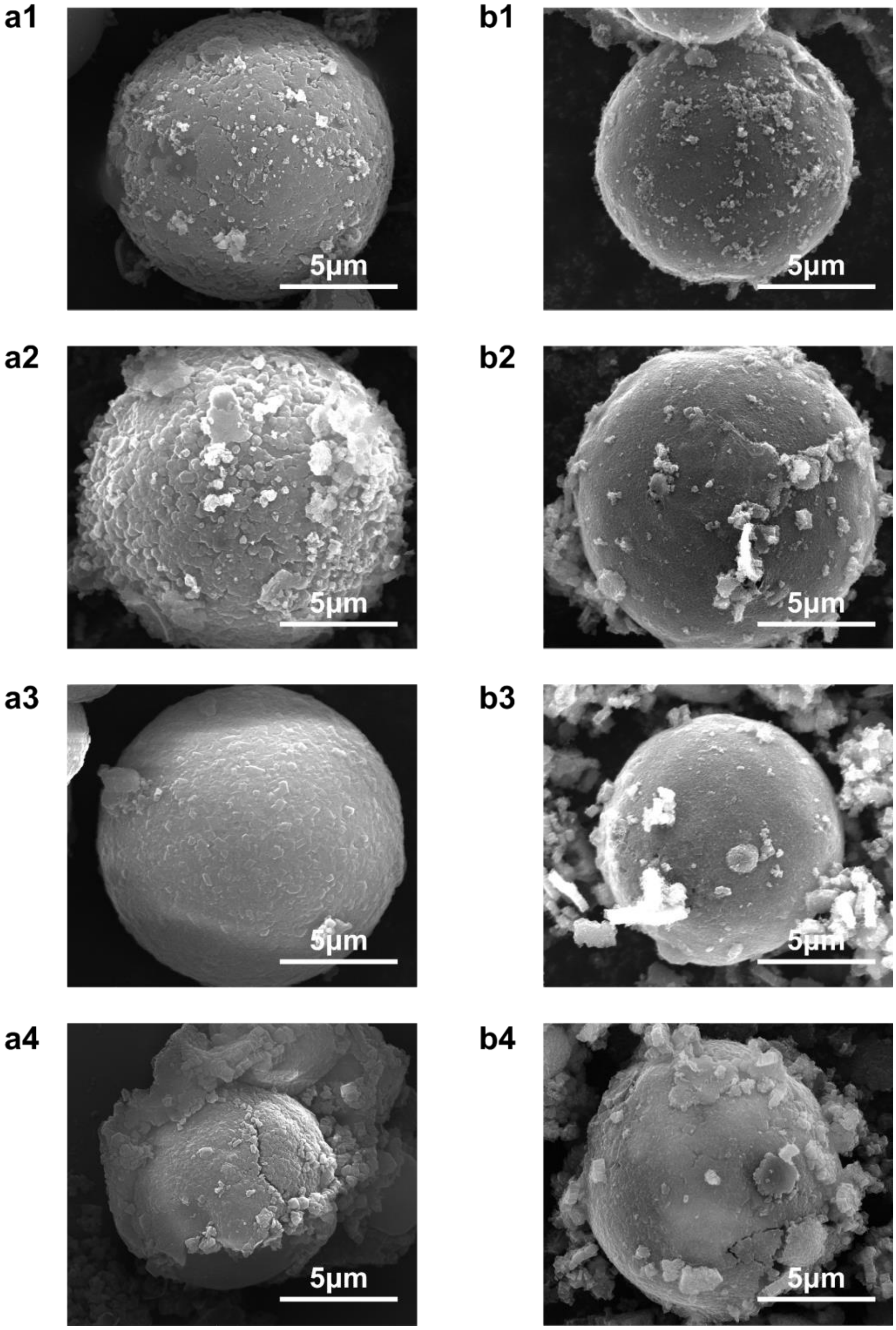

Fig. S3 a1-a4 SEM images of PMZ-1, PMZ-2, PMZ-3, and PMZ-4. **b1-b4** SEM images of HMCCo-1, HMCCo-2, HMCCo-3, and HMCCo-4

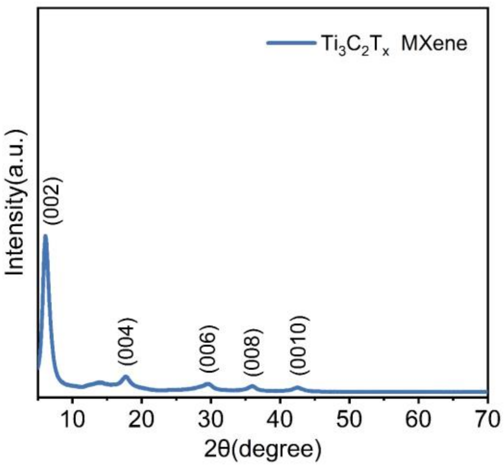

Fig. S4 XRD pattern of MXene

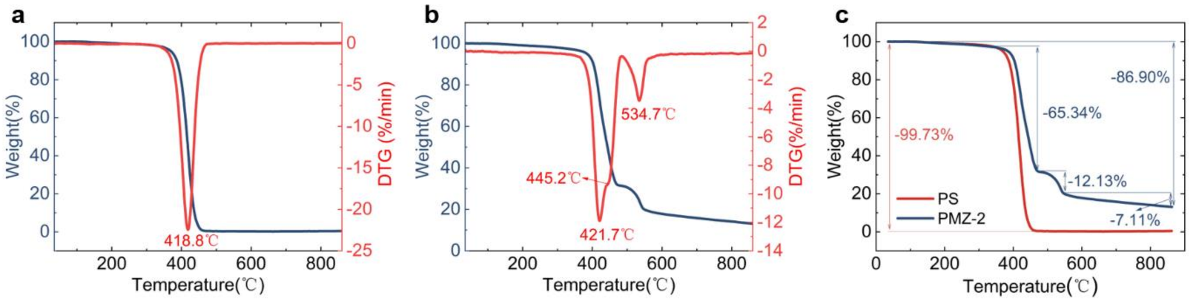

Fig.S5 Thermogravimetric analysis (TGA) and differential thermogravimetric analysis (DTG) of PS powder and PMZ-2 powder. **a** TGA and DTG of PS powders from room temperature to 880°C. **b** TGA and DTG of PMZ-2 powders from room temperature to 880°C. **c** TGA of PS and PMZ-2 powders from room temperature to 880°C. The test equipment was TG209 F3, tested under N_2 atmosphere with a heating rate of 10°C/min

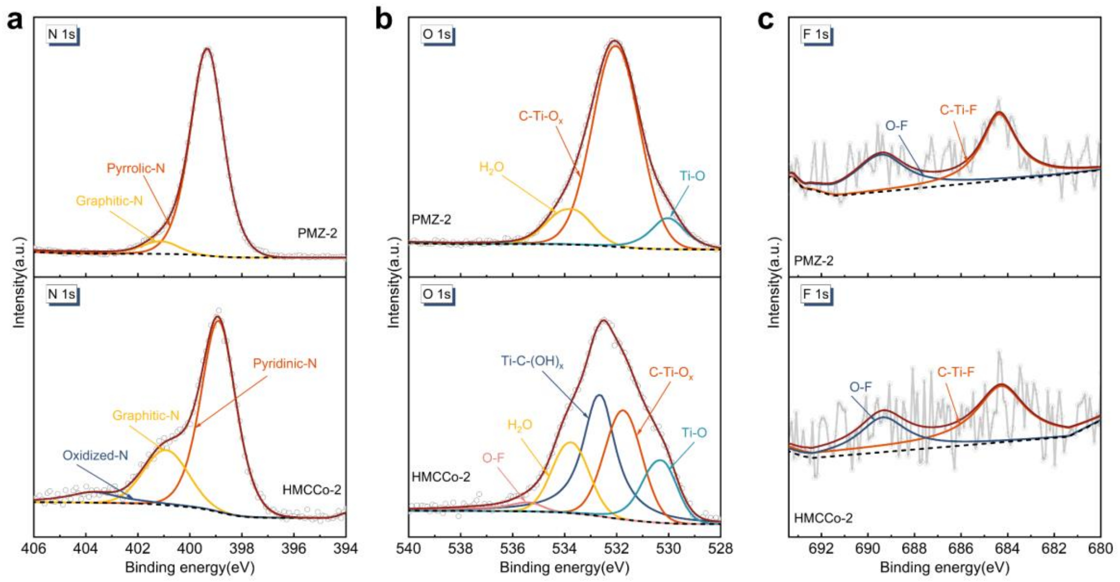

Fig. S6 a N 1s spectra of PMZ-2 and HMCCo-2. **b** O 1s spectra of PMZ-2 and HMCCo-2. **c** F 1s spectra of PMZ-2 and HMCCo-2

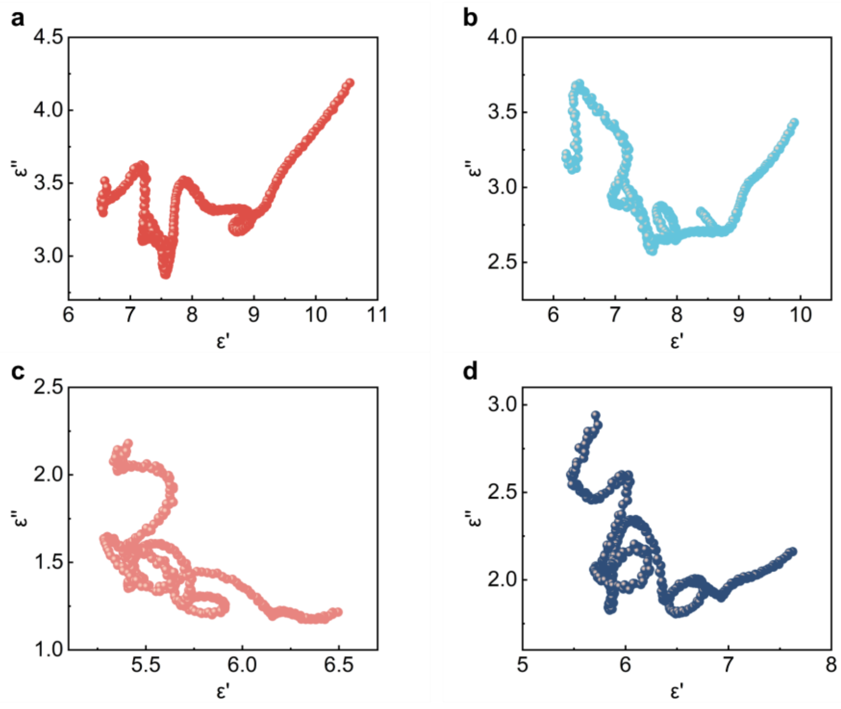

Fig. S7 The Cole-Cole semicircles of **a** HMCCo-1, **b** HMCCo-2, **c** HMCCo-3, and **d** HMCCo-4

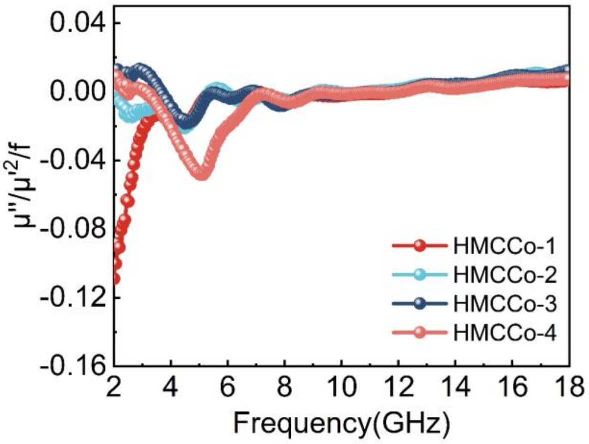

Fig. S8 Frequency-dependent $\mu''(\mu')^{-2}f^{-1}$ curves of HMCCo-1, HMCCo-2, HMCCo-3, and HMCCo-4

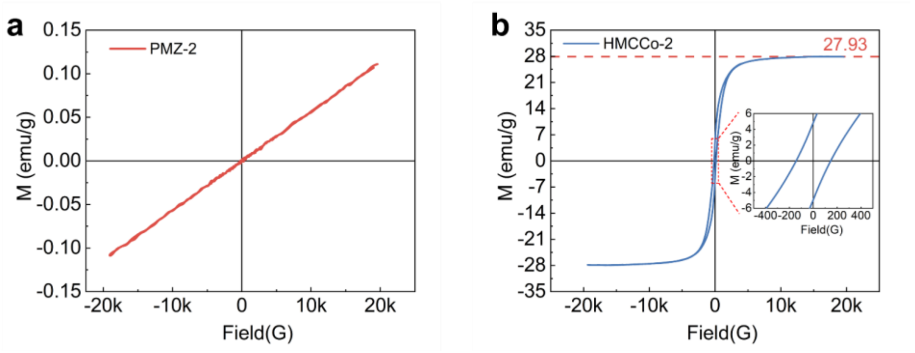

Fig. S9 Room-temperature hysteresis loop of **a** PMZ-2 and **b** HMCCo-2

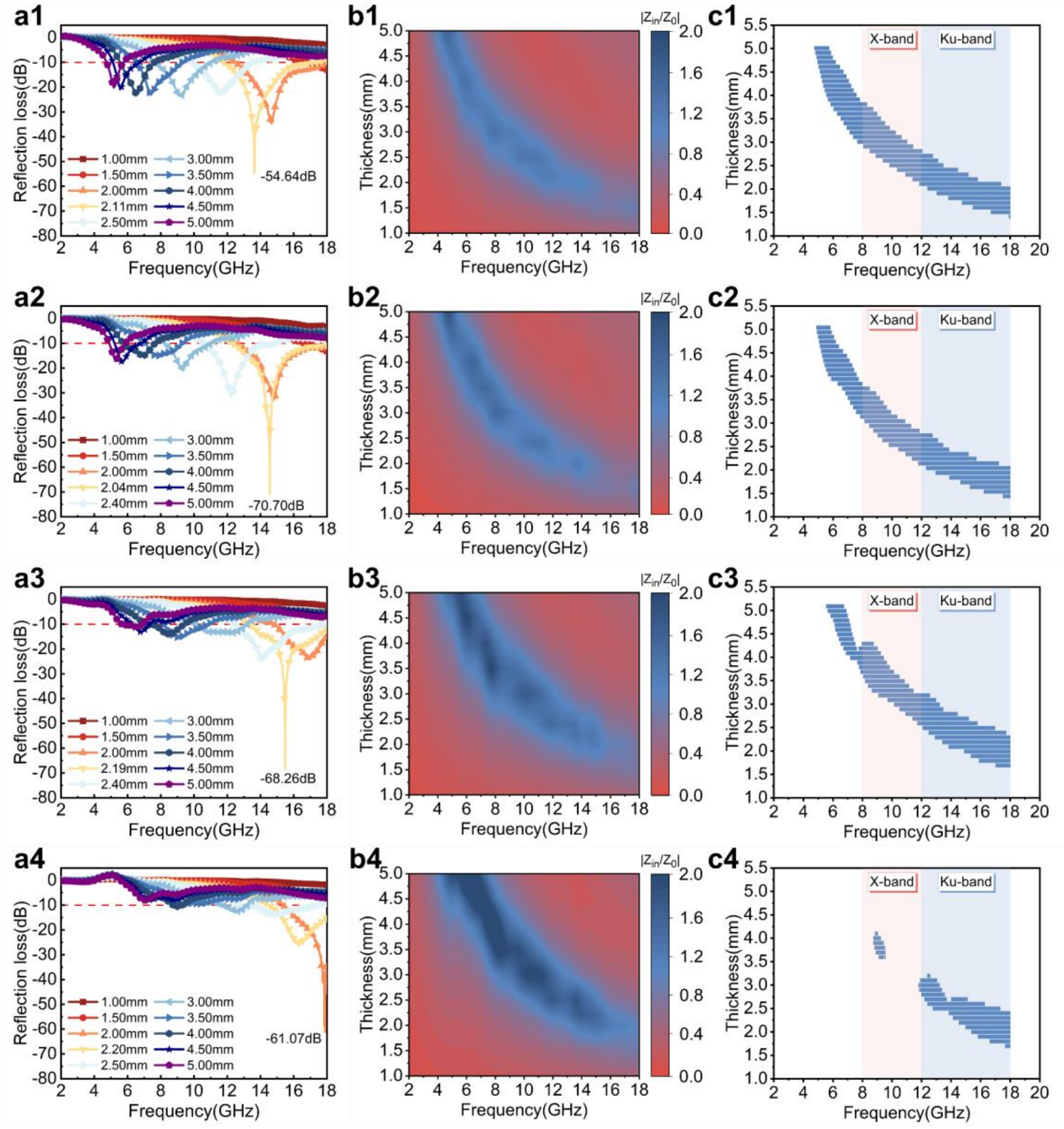

Fig. S10 2D plot of RL values for **a1** HMCCo-1, **a2** HMCCo-2, **a3** HMCCo-3, and **a4** HMCCo-4 with thicknesses ranging from 1-5mm. The characteristic impedance for **b1** HMCCo-1, **b2** HMCCo-2, **b3** HMCCo-3, and **b4** HMCCo-4. EAB bar charts for **c1** HMCCo-1, **c2** HMCCo-2, **c3** HMCCo-3, and **c4** HMCCo-4

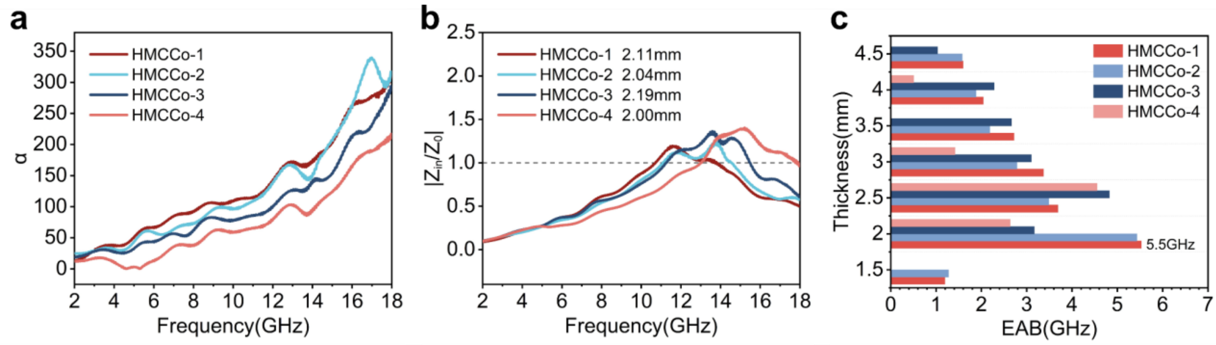

Fig. S11 **a** The attenuation constant, **b** characteristic impedance, and **c** EAB corresponding to 1.5-4.5 mm of the HMCCo series

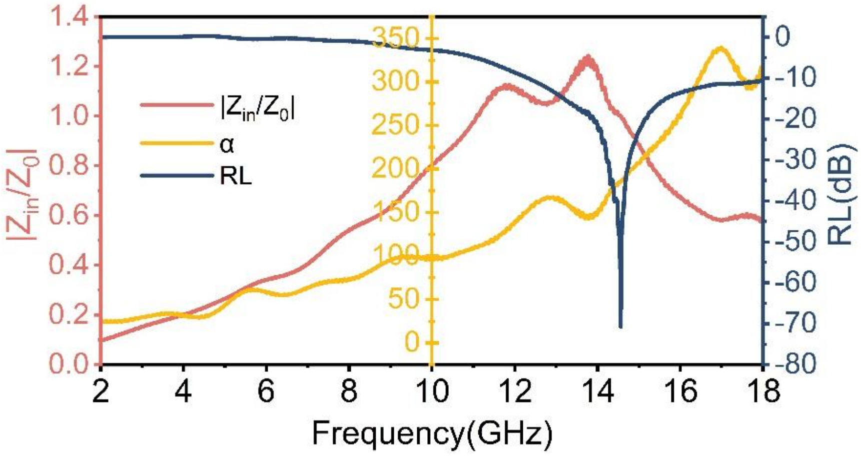

Fig. S12 $|Z_{in}/Z_0|$, attenuation constant, and RL value of HMCCo-2

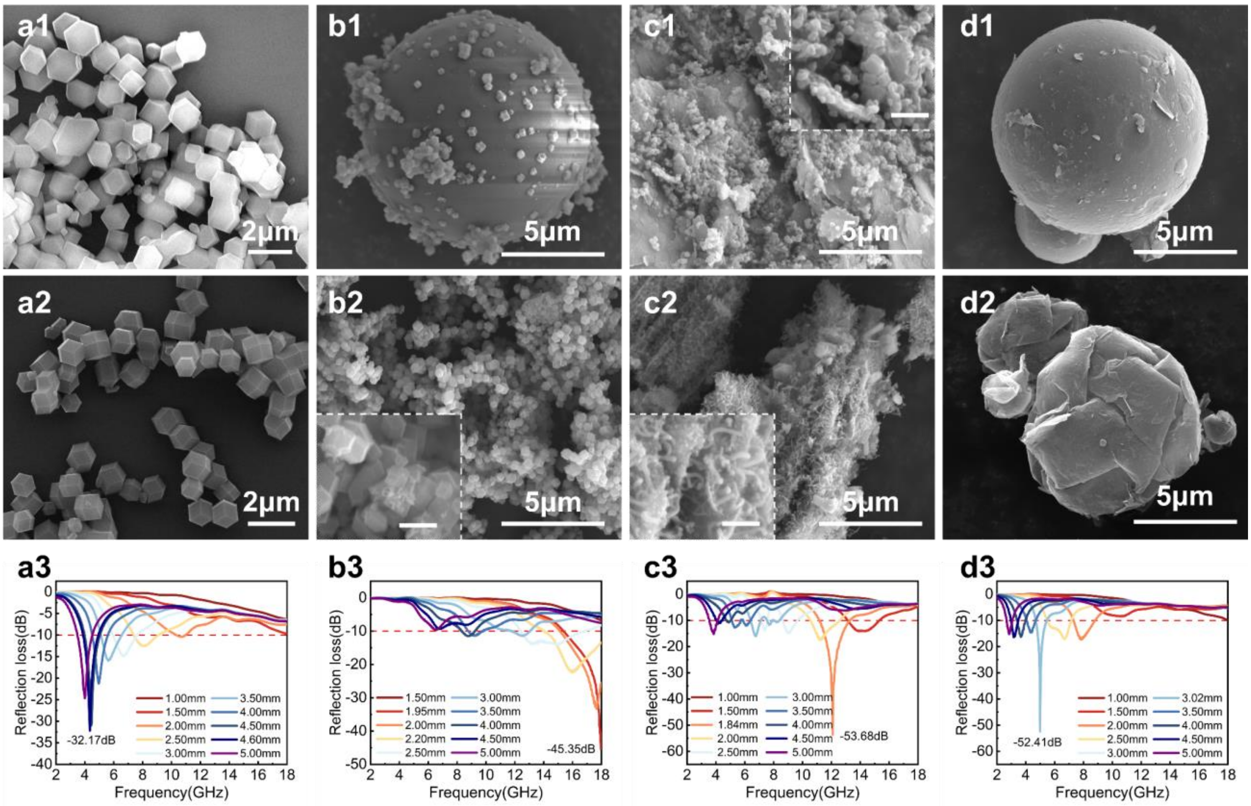

Fig. S13 SEM images of **a1** ZIF67, **b1** PS@ZIF67, **c1** MXene@ZIF67, and **d1** PS@MXene before calcination. SEM images of **a2** ZIF67-600, **b2** PS@ZIF67-600, **c2** MXene@ZIF67-600, **d2** and PS@MXene-600 after calcination at 600℃. RL values corresponding to 1-5 mm thickness for samples **a3** ZIF67-600, **b3** PS@ZIF67-600, **c3** MXene@ZIF67-600, and **d3** PS@MXene-600. The remaining scales in the Figure are all 1 μm

Figure S13 shows four sets of comparative experiments, in which factors such as sample ratio, preparation process, and reaction time before calcination were consistent with the parameters used for preparing the same product PMZ-2. The calcination temperature, holding time, and heating rate of the four groups of samples were consistent with the parameters for preparing HMCCo-2 under the same conditions. In addition, the method of preparing coaxial rings and the filler ratio are also consistent with the previous text.

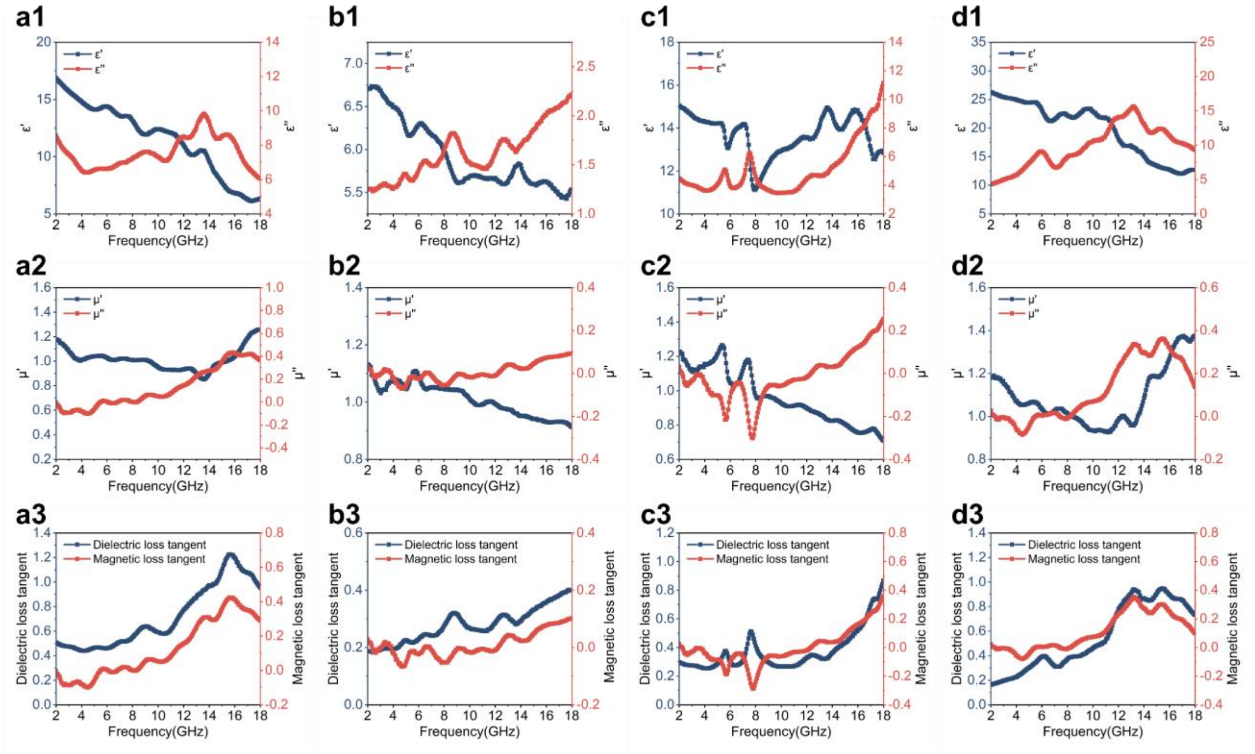

Fig. S14 a1 Real and imaginary parts of complex dielectric parameters, **a2** real and imaginary parts of complex permeability, and **a3** dielectric loss tangent and magnetic loss tangent for ZIF67-600. **b1** Real and imaginary parts of complex dielectric parameters, **b2** real and imaginary parts of complex permeability, and **b3** dielectric loss tangent and magnetic loss tangent for PS@ZIF67-600. **c1** Real and imaginary parts of complex dielectric parameters, **c2** real and imaginary parts of complex permeability, and **c3** dielectric loss tangent and magnetic loss tangent for MXene@ZIF67-600. **d1** Real and imaginary parts of complex dielectric parameters, **d2** real and imaginary parts of complex permeability, and **d3** dielectric loss tangent and magnetic loss tangent for PS@MXene-600

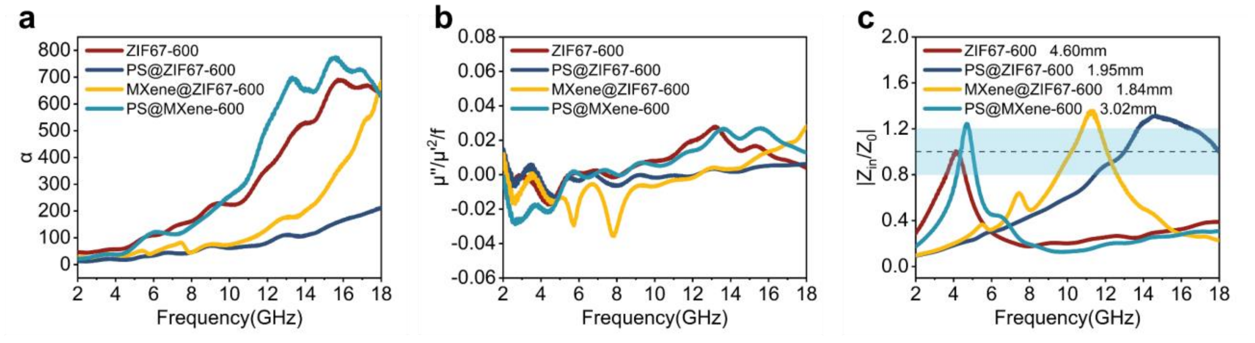

Fig. S15 a The attenuation constant curves and **b** $\mu''(\mu')^{-2}f^{-1}$ - Frequency curves of four samples. **c** $|Z_{in}/Z_0|$-value curves of four groups of samples at the corresponding thickness when RL value reaches the minimum

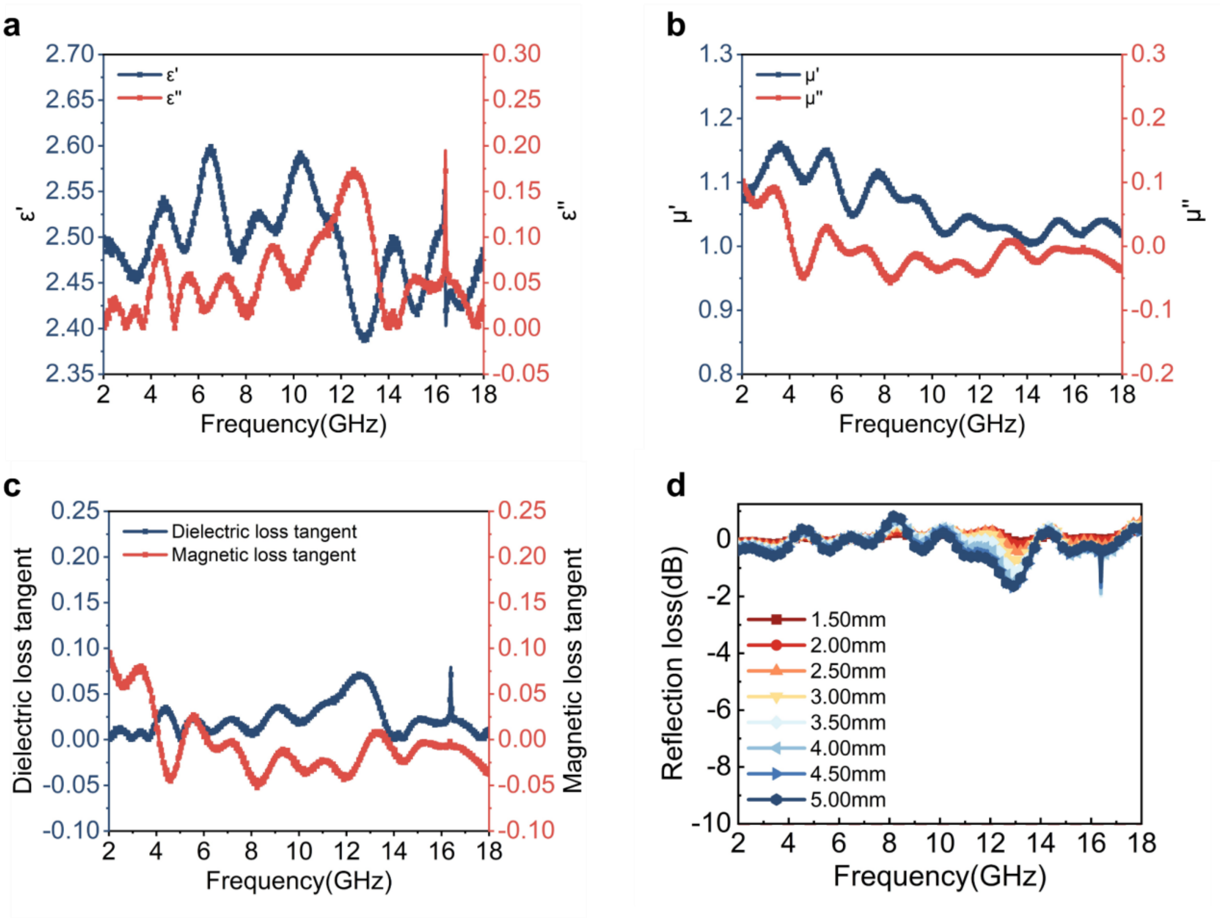

Fig. S16 Electromagnetic performance of sample PMZ-2. **a** Real and imaginary parts of complex dielectric parameters for PMZ-2. **b** Real and imaginary parts of complex permeability for PMZ-2. **c** Dielectric loss tangent and Magnetic loss tangent of PMZ-2. **d** RL values of PMZ-2 sample at a thickness of 1.5-5 mm

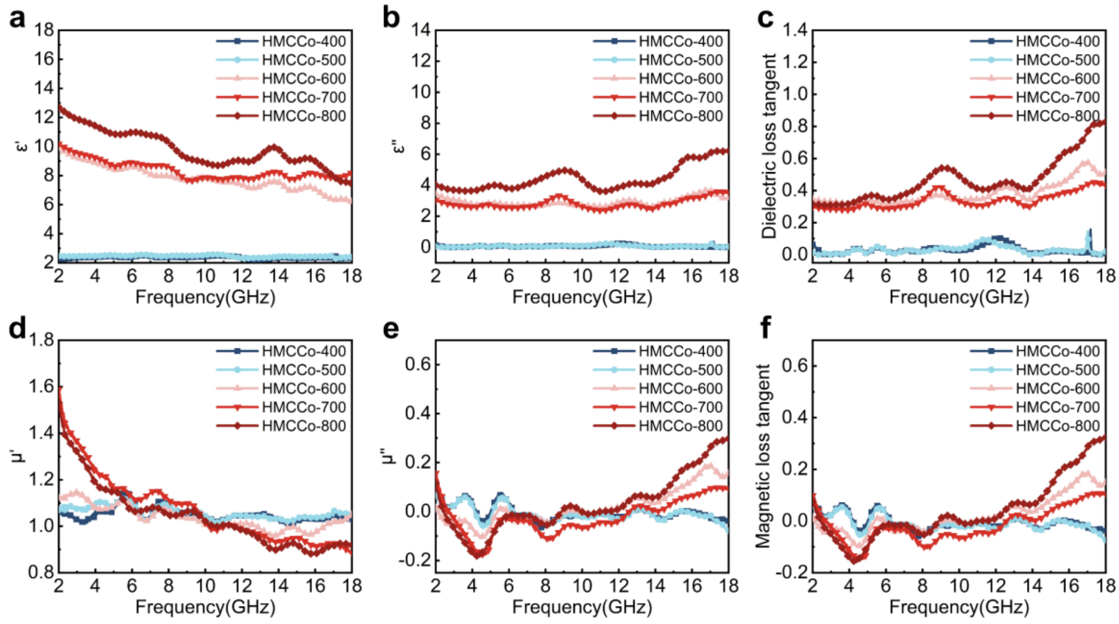

Fig. S17 The **a** complex permittivity real parts, **b** complex permittivity imaginary parts, **c** dielectric loss tangent, **d** complex permeability real parts, **e** complex permeability imaginary parts, and **f** magnetic loss tangent curves of HMCCo obtained at different temperatures

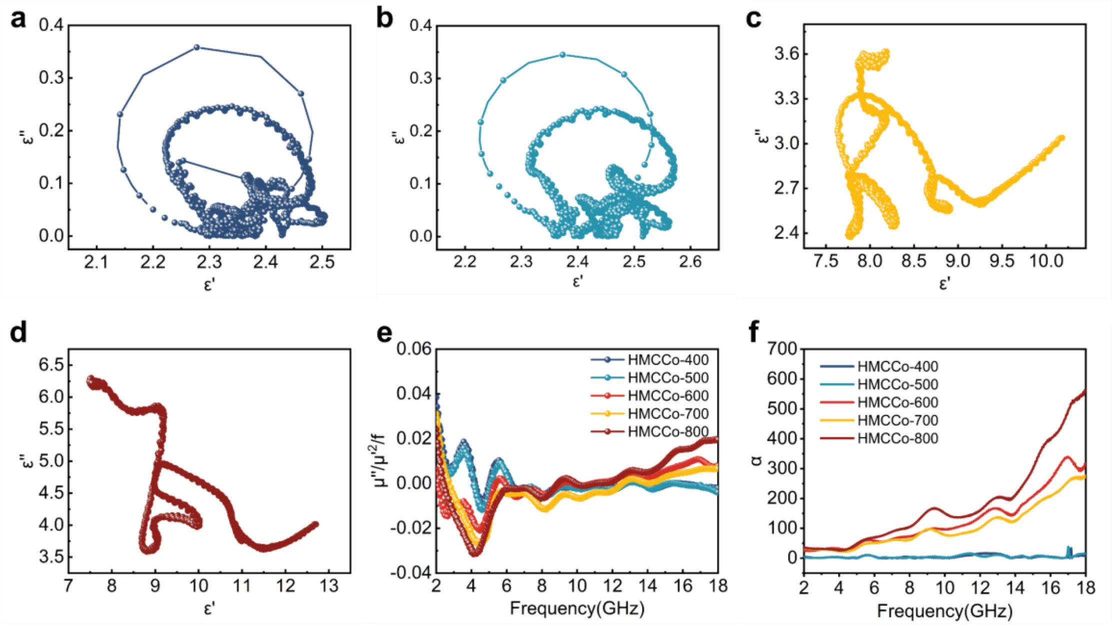

Fig. S18 The Cole-Cole plots of **a** HMCCo-400, **b** HMCCo-500, **c** HMCCo-700, and **d** HMCCo-800. **e** $\mu''(\mu')^{-2}f^{-1}$ values and **f** attenuation constant of HMCCo series

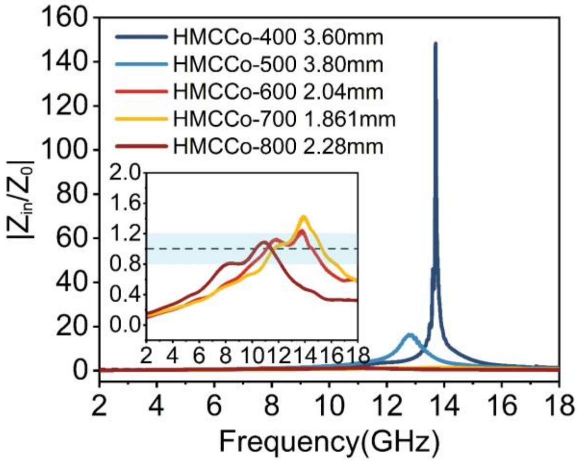

Fig. S19 The characteristic impedance values of HMCCo-400~HMCCo-800 under matching thickness

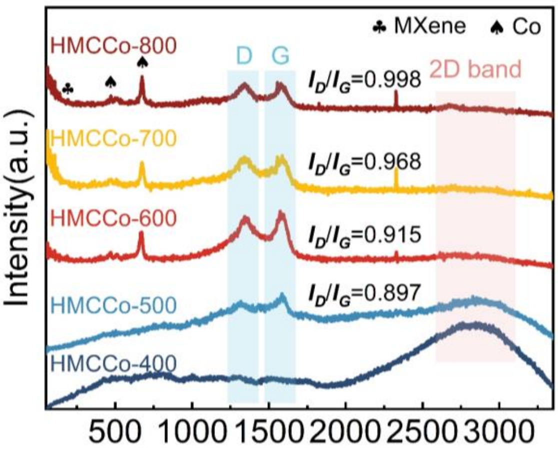

Fig. S20 Raman spectra of HMCCo-400, HMCCo-500, HMCCo-600, HMCCo-700, and HMCCo-800

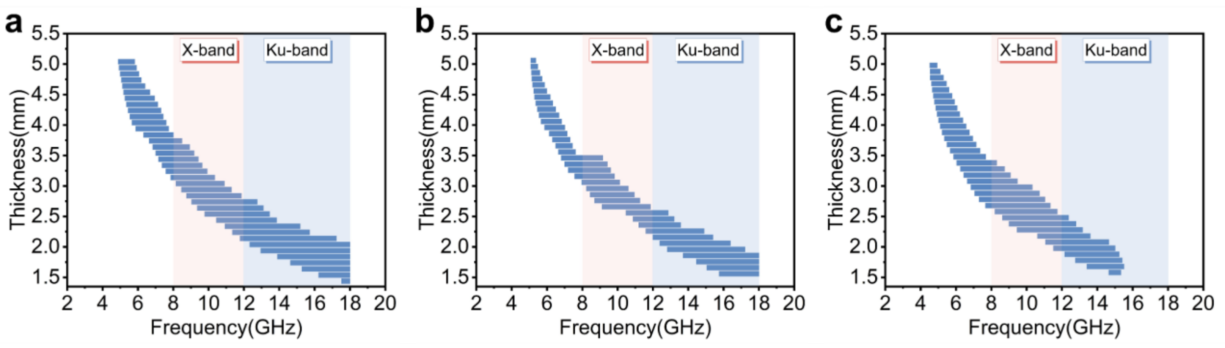

Fig. S21 EAB of **a** HMCCo-600, **b** HMCCo-700, and **c** HMCCo-800

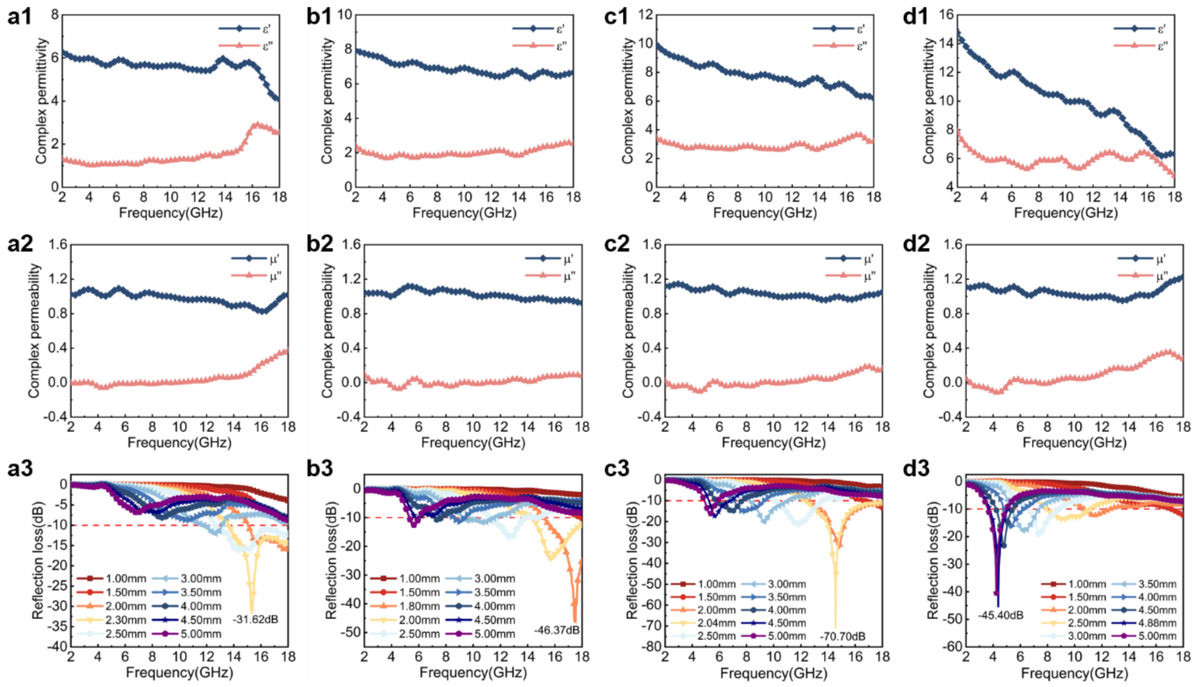

Fig. S22 a1 Complex permittivity, **a2** complex permeability and **a3** RL curves at 5 wt% filler ratio. **b1** Complex permittivity, **b2** complex permeability and **b3** RL curves at 10 wt% filler ratio. **c1** Complex permittivity, **c2** complex permeability and **c3** RL curves at 15 wt% filler ratio. **d1** Complex permittivity, **d2** complex permeability and **d3** RL curves at 20 wt% filler ratio

As shown in the Fig. S22, with the increase of mass ratio, the real part of complex dielectric constant is increasing, and the electromagnetic wave absorption performance first increases and then decreases, and the minimum reflection loss value is minimized at 15 wt%, which is -70.70 dB. When the filler ratio reaches 20 wt%, the complex dielectric constant of the material increases dramatically. According to the free electron theory, an excessively high dielectric constant will result in more incident electromagnetic waves being reflected by the absorber, which is detrimental to the dissipation of electromagnetic waves. In summary, we believe that the dielectric constant and magnetic permeability of the material are maintained at an appropriate level at a filler ratio of 15 wt%, so we uniformly use a filler ratio of 15 wt% in our study.

Table S1 List of instructions for preparing sample labels

Materials	Mass ratio of PS@MXene and $Co(NO_3)_2 \cdot 6H_2O$	Labels	Calcination temperature	Labels
PS@MXene	/	PM	600°C	HM
PS@MXene@ZIF67	When the load capacity of ZIF67 is different			
	1:1	PMZ-1	600°C	HMCCo-1
	1:2	PMZ-2	600°C	HMCCo-2
	1:3	PMZ-3	600°C	HMCCo-3
	1:4	PMZ-4	600°C	HMCCo-4
	When the calcination temperature is different			
	1:2	PMZ-2	400°C	HMCCo-400
	1:2	PMZ-2	500°C	HMCCo-500
	1:2	PMZ-2	600°C	HMCCo-600
	1:2	PMZ-2	700°C	HMCCo-700
	1:2	PMZ-2	800°C	HMCCo-800

Table S2 Comparison Table of Electromagnetic Wave Absorption Performance of MXene/CNTs System Materials

Components	RL_{min}(dB)	d(mm)	EAB(GHz)	Refs.
MXene@CNTs	-48.80	1.72	5.44	[S1]
MXene/CNTs/PI	-50.03	1.70	5.60	[S2]
MXene/CNTs/Fe_3O_4	-40.10	2.00	5.80	[S3]
MXene/CNTs/Co	-85.80	1.40	6.10	[S4]
CNTs/Ti_3C_2-SA	-40.00	3.95	4.20	[S5]
MXene@NiCo@NCNTs	-55.30	2.10	4.30	[S6]
NiCo/TiC/TiO/CNTs	-51.98	1.90	7.76	[S7]
MXene/ZIF67-C@Co/MXene	-50.50	4.00	5.80	[S8]
MXene/ZIF67-MXene/CoNi/N-CNTs	-52.64	3.80	1.60	[S9]
MXene/CNTs/Ni	-56.40	2.40	3.95	[S10]
Co/CNTs-MXene@CF(Co-ZIF)	-61.41	2.52	5.04	[S11]
MXene/Ni/N-CNTs	-57.78	1.49	3.44	[S12]
MXene/CNTs	-52.90	1.55	4.46	[S13]
MXene/CNTs@Fe-Co	-52.56	2.50	2.16	[S14]
MXene/CoNi/CNTs	-51.60	1.60	4.50	[S15]
MXene/CNTs	-45.00	2.70	4.90	[S16]
MXene/CNFs/CoNi/TiO_2	-54.60	1.76	4.00	[S17]
MXene/Co-ZIF67	-60.09	2.70	9.30	[S18]
HMCCo-600 or HMCCo-2	-70.70	2.04	5.67	This work
HMCCo-700	-63.25	1.861	4.38	This work
HMCCo-800	-62.91	2.28	3.70	This work

Supplementary References

[S1] K. Hu, H. Wang, W. Cheng, Y. Rao, L. Pan et al., Lightweight and robust $Ti_3C_2T_x$/carbon nanotubes foam with tuneable and highly efficient microwave absorption performance. Ceram. Int. **48**(12), 31129-31137 (2022). https://doi.org/10.1016/j.ceramint.2022.05.325

[S2] Y. Cui, K. Yang, F. Zhang, Y. Lyu, Q. Zhang et al., Ultra-light MXene/CNTs/PI aerogel with neat arrangement for electromagnetic wave absorption and photothermal

conversion. Compos. Part A Appl. Sci. Manuf. **158**, 106986 (2022). https://doi.org/10.1016/j.compositesa.2022.106986

[S3] C. Zhang, Z. Wu, C. Xu, B. Yang, L. Wang et al., Hierarchical $Ti_3C_2T_x$ MXene/Carbon Nanotubes Hollow Microsphere with Confined Magnetic Nanospheres for Broadband Microwave Absorption. Small **18**(3), 2104380 (2022). https://doi.org/10.1002/smll.202104380

[S4] Z. Xiang, Y. Shi, X. Zhu, L. Cai, W. Lu, Flexible and Waterproof 2D/1D/0D Construction of MXene-Based Nanocomposites for Electromagnetic Wave Absorption, EMI Shielding, and Photothermal Conversion. Nano-Micro Lett. **13**(10), 150 (2021). https://doi.org/10.1007/s40820-021-00673-9

[S5] X. Liu, N. Chai, Z. Yu, H. Xu, X. Li et al., Ultra-light, high flexible and efficient CNTs/Ti_3C_2-sodium alginate foam for electromagnetic absorption application. J. Mater. Sci. Technol. **35**(12), 2859-2867 (2019). https://doi.org/10.1016/j.jmst.2019.07.019

[S6] J. Cheng, B. Liu, Y. Wang, H. Zhao, Y. Wang, Y. Growing CoNi nanoalloy@N-doped carbon nanotubes on MXene sheets for excellent microwave absorption. J. Mater. Sci. Technol. **130**(35), 157-165 (2022). https://doi.org/10.1016/j.jmst.2022.05.013

[S7] T. Hou, Z. Jia, Y. Dong, X. Liu, G. Wu, Layered 3D structure derived from MXene/magnetic carbon nanotubes for ultra-broadband electromagnetic wave absorption. Chem. Eng. J. **431**, 133919 (2022). https://doi.org/10.1016/j.cej.2021.133919

[S8] Z. Zou, M. Ning, Z. Lei, X. Zhuang, G. Tan et al., 0D/1D/2D architectural Co@C/MXene composite for boosting microwave attenuation performance in 2-18 GHz. Carbon. **193**, 182-194 (2022). https://doi.org/10.1016/j.carbon.2022.03.017

[S9] J. Zhou, F. Guo, J. Luo, G. Hao, G. Liu, Designed 3D heterostructure with 0D/1D/2D hierarchy for low-frequency microwave absorption in the S-band. J. Mater. Chem. C. **10**(4), 1470-1478 (2022). https://doi.org/10.1039/D1TC04881C

[S10] X. Li, W. You, C. Xu, L. Wang, L. Yang et al., 3D Seed-Germination-Like MXene with In Situ Growing CNTs/Ni Heterojunction for Enhanced Microwave Absorption via Polarization and Magnetization. Nano-Micro Lett. **13**(10), 157(2021). https://doi.org/10.1007/s40820-021-00680-w

[S11] B. Wang, S. Li, F. Huang, S. Wang, H. Zhang et al., Construction of multiple electron transfer paths in 1D core-shell hetetrostructures with MXene as interlayer enabling efficient microwave absorption. Carbon **187**, 56-66 (2022). https://doi.org/10.1016/j.carbon.2021.10.080

[S12] J. Zhou, G. Zhang, J. Luo, Y. Hu, G. Hao et al., A MOFs-derived 3D superstructure nanocomposite as excellent microwave absorber. Chem. Eng. J. **426**, 130725 (2021). https://doi.org/10.1016/j.cej.2021.130725

[S13] X. Li, X. Yin, M. Han, C. Song, H. Xu et al., Ti_3C_2 MXenes modified with in situ grown carbon nanotubes for enhanced electromagnetic wave absorption properties. J. Mater. Chem. C. **5**(16), 468-474 (2017). https://doi.org/10.1039/C6TC05226F

[S14] Y. Yue, Y. Wang, X. Xu, C. Wang, Z. Yao et al., In-situ growth of bamboo-shaped carbon nanotubes and helical carbon nanofibers on $Ti_3C_2T_x$ MXene at ultra-low temperature for enhanced electromagnetic wave absorption properties. Ceram. Int. **48**(5), 6338-6346 (2022). https://doi.org/10.1016/j.ceramint.2021.11.176

[S15] F. Wu, Z. Liu, J. Wang, T. Shah, P. Liu et al., Template-free self-assembly of MXene and CoNi-bimetal MOF into intertwined one-dimensional heterostructure and its

microwave absorbing properties. Chem. Eng. J. **422**, 130591 (2021). https://doi.org/10.1016/j.cej.2021.130591

[S16] Y. Cui, F. Wu, J. Wang, Y. Wang, T. Shah et al., Three dimensional porous MXene/CNTs microspheres: Preparation, characterization and microwave absorbing properties. Compos. Part A Appl. Sci. Manuf. 145, 106378 (2021). https://doi.org/10.1016/j.compositesa.2021.106378

[S17] Z. Liu, J. Chen, M. Que, H. Zheng, L. Yang et al., 2D $Ti_3C_2T_x$ MXene/MOFs composites derived CoNi bimetallic nanoparticles for enhanced microwave absorption. Chem. Eng. J. **450**, 138442 (2022). https://doi.org/10.1016/j.cej.2022.138442

[S18] X. Han, Y. Huang, L. Ding, Y. Song, T. Li et al., $Ti_3C_2T_x$ MXene Nanosheet/Metal-Organic Framework Composites for Microwave Absorption. ACS Appl. Nano Mater. **4**(1), 691-701 (2021). https://doi.org/10.1021/acsanm.0c02983
